# MOF‐on‐MOF‐Derived Hollow Co_3_O_4_/In_2_O_3_ Nanostructure for Efficient Photocatalytic CO_2_ Reduction

**DOI:** 10.1002/advs.202300797

**Published:** 2023-04-21

**Authors:** Cheng Han, Xiaodeng Zhang, Shengsheng Huang, Yue Hu, Zhi Yang, Ting‐Ting Li, Qipeng Li, Jinjie Qian

**Affiliations:** ^1^ Key Laboratory of Carbon Materials of Zhejiang Province College of Chemistry and Materials Engineering Wenzhou University Wenzhou Zhejiang 325000 P. R. China; ^2^ College of Chemistry and Chemical Engineering Zhaotong University Zhaotong Yunnan 657000 P. R. China; ^3^ School of Materials Science and Chemical Engineering Ningbo University Ningbo Zhejiang 315211 P. R. China

**Keywords:** bimetallic oxide, CO_2_ reduction, metal‐organic framework, MOF‐on‐MOF heterostructure, photocatalyst

## Abstract

The photocatalytic transformation of carbon dioxide (CO_2_) into carbon‐based fuels or chemicals using sustainable solar energy is considered an ideal strategy for simultaneously alleviating the energy shortage and environmental crises. However, owing to the low energy utilization of sunlight and inferior catalytic activity, the conversion efficiency of CO_2_ photoreduction is far from satisfactory. In this study, a MOF‐derived hollow bimetallic oxide nanomaterial is prepared for the efficient photoreduction of CO_2_. First, a unique ZIF‐67‐on‐InOF‐1 heterostructure is successfully obtained by growing a secondary Co‐based ZIF‐67 onto the initial InOF‐1 nanorods. The corresponding hollow counterpart has a larger specific surface area after acid etching, and the oxidized bimetallic H‐Co_3_O_4_/In_2_O_3_ material exhibits abundant heterogeneous interfaces that expose more active sites. The energy band structure of H‐Co_3_O_4_/In_2_O_3_ corresponds well with the photosensitizer of [Ru(bpy)_3_]Cl_2_, which results in a high CO yield of 4828 ± 570 µmol h^−1^ g^−1^ and stable activity over a consecutive of six runs, demonstrating adequate photocatalytic performance. This study demonstrates that the rational design of MOF‐on‐MOF heterostructures can completely exploit the synergistic effects between different components, which may be extended to other MOF‐derived nanomaterials as promising catalysts for practical energy conversion and storage.

## Graphical abstract

In this article, the hollow counterpart of H‐ZIF‐67‐on‐InOF‐1 presents a larger specific surface area after being acid‐etched, and its corresponding bimetallic oxide of H‐Co_3_O_4_/In_2_O_3_ owns abundant heterogeneous interfaces, which show a satisfactory photocatalytic CO_2_ reduction.

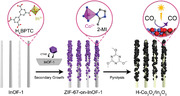

## 1 Introduction

Global warming mainly originates from the excessive emission of carbon dioxide (CO_2_) caused by the rapid consumption of fossil fuels, resulting in an increasing concentration of CO_2_ in the atmosphere.^[^ ^1^, ^2^, ^3^ ^]^ In this context, the conversion of CO_2_ into environmentally friendly chemicals is considered a promising research direction worldwide, thereby alleviating several environmental issues.^[^ ^4^, ^5^, ^6^, ^7^ ^]^ However, it may seem paradoxical to input massive carbon‐based fuels to efficiently cleave the highly stable C=O bonds in CO_2_. Therefore, the reduction of CO_2_ emissions requires sustainable energy from green energy sources rather than burning fossil fuels. Numerous efforts have been made to capture, separate, store, and utilize CO_2_.^[^ ^8^, ^9^, ^10^, ^11^ ^]^ Therefore, the efficient photocatalytic reduction of CO_2_ using renewable and clean solar energy has received extensive attention.

Under these circumstances, enhancing the light‐harvesting efficiency of catalysts is the main method to improve their photocatalytic activity. Because visible light accounts for ≈50% of the total solar energy, it is crucial to develop high‐performance visible‐light‐responsive photocatalysts. To date, various inorganic metal oxide nanomaterials, such as ZnO, TiO_2_, In_2_O_3_, and Co_3_O_4_, have been demonstrated as potential photo‐responsive species.^[^ ^12^, ^13^, ^14^ ^]^ However, all of these reported single semiconductor catalysts exhibit unsatisfactory catalytic activity, mainly because their wide bandgap properties allow only a small fraction of sunlight to be utilized.^[^ ^15^, ^16^ ^]^ In contrast, CO_2_ molecules can only be activated by adsorption onto the catalyst surface. Therefore, photocatalysts that can efficiently capture guest molecules can accelerate the CO_2_ reduction reaction (CO_2_RR).^[^ ^17^, ^18^, ^19^, ^20^ ^]^ Therefore, ideal photocatalysts are intrinsically endowed with excellent activity, selectivity, and stability, and should simultaneously exhibit sufficient visible‐light absorption capacity as well as a large CO_2_ sorption ability.

Porous metal‐organic frameworks (MOFs), which are an emerging subclass of porous coordination polymers, have been extensively used in gas adsorption and separation, drug delivery, and heterogeneous catalysis.^[^ ^21^, ^22^, ^23^, ^24^, ^25^, ^26^ ^]^ Owing to their high porosity, large surface area, and tuneable nanostructure, MOF materials have been demonstrated to be favorable precursors for the facile preparation of porous metal oxides.^[^ ^27^, ^28^ ^]^ Among them, MOF‐derived Co_3_O_4_ photocatalysts have been exploited as potential candidates for photocatalytic CO_2_ conversion.^[^ ^29^, ^30^ ^]^ However, the availability of most reported cobalt‐based catalysts is severely limited owing to their modest CO_2_ adsorption and inferior visible‐light utilization efficiency.^[^ ^31^ ^]^ Therefore, it is crucial to rationally design an efficient and robust Co_3_O_4_ photocatalyst capable of strongly capturing CO_2_ molecules and activating stable non‐polar bonds.

**Scheme 1:**
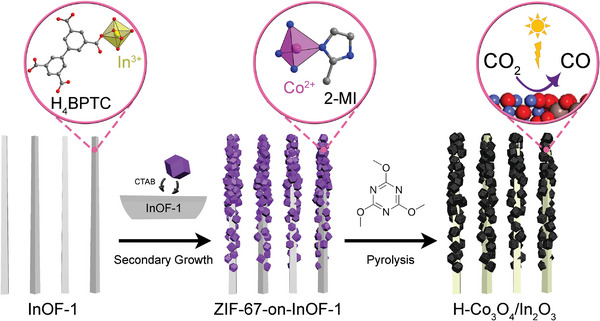
Stepwise fabrication of MOF‐on‐MOF‐derived hollow bimetallic photocatalyst H‐Co_3_O_4_/In_2_O_3_ for the CO_2_RR.

In this study, secondary Co‐based ZIF‐67 particles are grown on the initial In‐based InOF‐1 nanorods to obtain a unique MOF‐on‐MOF heterostructure, as illustrated in **Scheme** **1**, denoted as ZIF‐67‐on‐InOF‐1. Using acid etching, followed by low‐temperature oxidation in air, the formation of a hollow bimetallic oxide nanomaterial, H‐Co_3_O_4_/In_2_O_3_, is achieved. H‐Co_3_O_4_/In_2_O_3_ has an interesting conjunction containing p‐type Co_3_O_4_ and porous In_2_O_3_, in which these active cobalt phases are strongly anchored onto the supporting In_2_O_3_, exposing abundant heterogeneous interfaces. In this case, the as‐synthesized MOF‐on‐MOF‐derived hollow bimetallic oxide photocatalyst had a lower activation barrier with high CO selectivity and sufficient structural stability for the CO_2_RR, which was considerably higher than those of the control samples.

## 2 Results and Discussion

**Figure 1:**
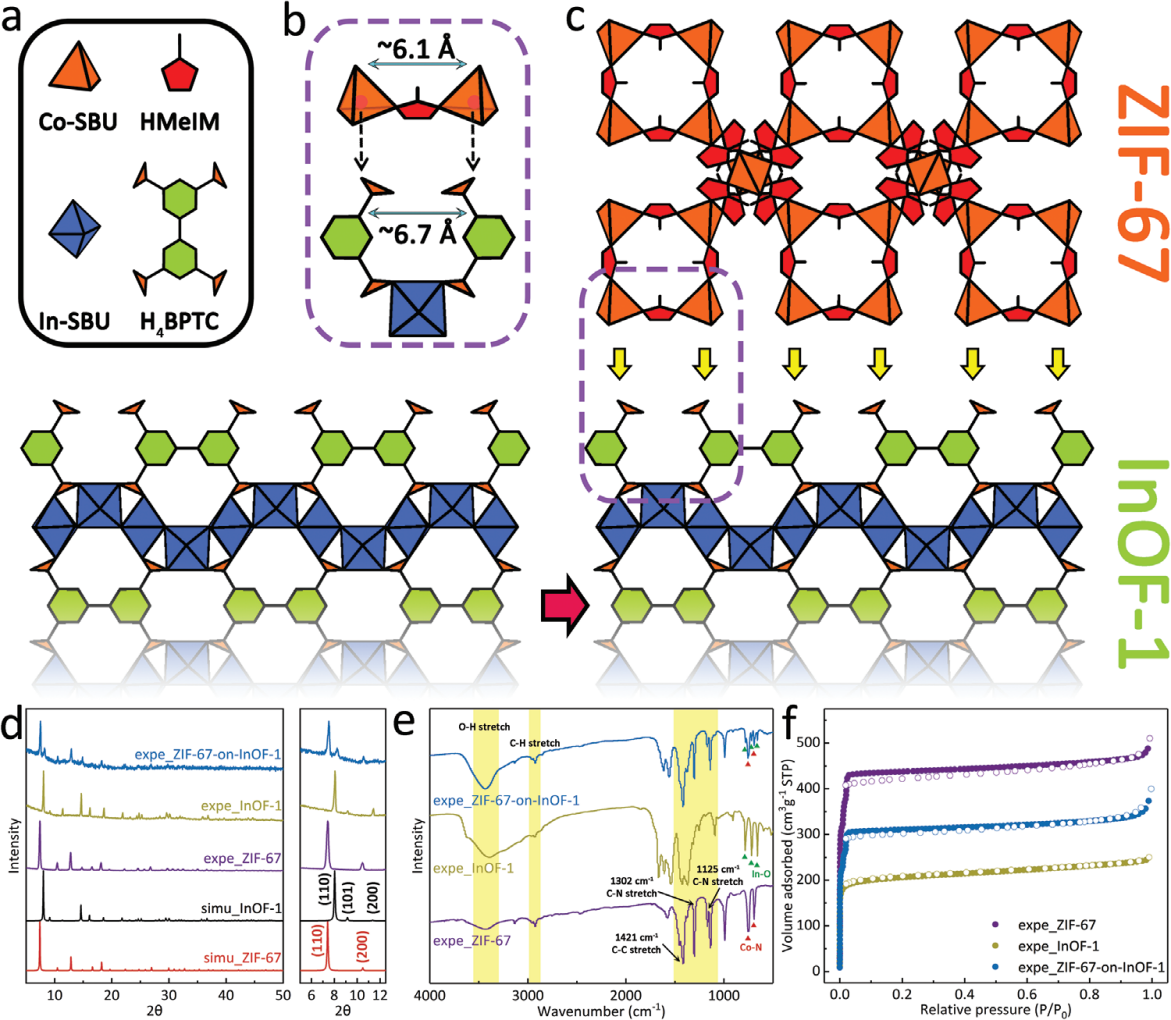
a) Diagram of inorganic SBUs and organic linkers; b) Lattice comparison; c) Schematic of the fabrication of ZIF‐67‐on‐InOF‐1; d–f) PXRD patterns, FT‐IR spectra, and N_2_ isotherms of ZIF‐67, InOF‐1, and ZIF‐67‐on‐InOF‐1, respectively.

Using crystal engineering, a unique MOF‐on‐MOF heterostructure, ZIF‐67‐on‐InOF‐1, was obtained. In contrast to the invariable Co—N bonds in ZIF‐67, the six‐coordinate In(III) centers produce an octahedral structure in InOF‐1, where four O atoms are from the BPTC^4−^ ligand and two O atoms are from the solvent (Figures S1–S6, Tables S1 and S2, Supporting Information). In this case, the secondary building units (SBUs) of ZIF‐67 and InOF‐1 are represented as tetrahedra (orange) and octahedra (blue), respectively, and the organic ligands are simplified into polygons, as illustrated in **Figure** **1**a. The higher electronegativity of the O atoms in InOF‐1 can strongly attract Co(II) ions; therefore, they can be completely dispersed between the pores and surfaces of InOF‐1 with polyvinylpyrrolidone (PVP, Figure S7, Supporting Information). As shown in Figure 1b,c, Figure S8 (Supporting Information), the spacing between two adjacent O atoms was calculated to be 6.7 Å for InOF‐1, which is similar to that for ZIF‐67 (6.1 Å). This is a key prerequisite for the efficient growth of secondary ZIF‐67 on the surface of InOF‐1 after the addition of HMeIM. For ZIF‐67‐on‐InOF‐1, the characteristic powder X‐ray diffraction (PXRD) peak at 7.42° corresponded with the (110) crystal plane of ZIF‐67, and the peak at 8.02° was attributed to the (110) plane of InOF‐1 (Figure 1d). Figure 1e and Figure S9 (Supporting Information) show the FT‐IR spectra with a series of peaks between 650 and 800 cm^−1^ from the Co—N/In—O bonds in both MOFs, and two strong peaks at 1125 and 1302 cm^−1^ attributed to C–N stretching in ZIF‐67. In addition, Raman spectroscopy was performed to verify the formation of the MOF‐on‐MOF heterostructure, as shown in Figure S10 (Supporting Information). Finally, the N_2_ isotherms revealed that the adsorption capacity of InOF‐1 was significantly increased by increasing the ZIF‐67 content, and the micropore content of InOF‐1 also increased (Figure 1f and Figure S11, Table S3, Supporting Information).

**Figure 2:**
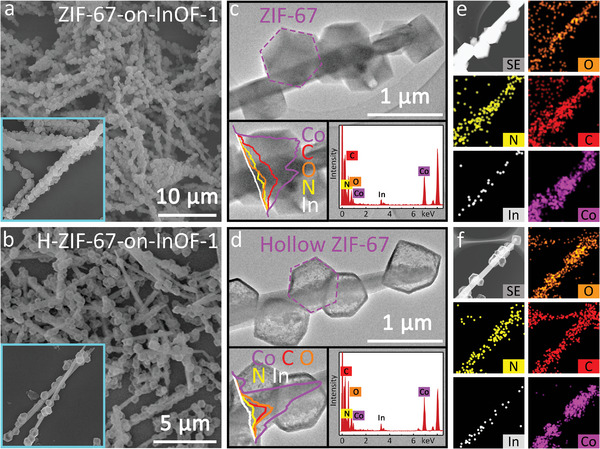
a,b) SEM images, c,d) TEM images, line‐scan profiles, and EDS spectra, e,f) HAADF‐STEM and EDS elemental mapping images for ZIF‐67‐on‐InOF‐1 and its etched hollow counterpart, respectively.

Scanning transmission electron microscopy (STEM) clearly characterized the successful synthesis of a heterostructure with ultra‐long InOF‐1 nanorods completely encapsulated by ZIF‐67 particles (**Figure** **2**a, Figure S12, Supporting Information). When the powders were well‐dispersed in ethanol and centrifuged, a colorless and transparent mixture was observed, confirming the strong interfacial forces between the two types of MOFs (Figure S13, Supporting Information). To increase the specific surface area and mass transfer, the obtained composite was further chemically etched with cyanuric acid (Figure 2b and Figures S14–S15, Supporting Information).^[^ ^32^ ^]^ Although partial detachment was observed owing to the high temperature and acid etching, the hierarchical nanostructure of H‐ZIF‐67‐on‐InOF‐1 was maintained. As shown in the inset in Figure 2c, the Co signal at both ends of ZIF‐67 was significantly stronger, and C, N, and O were also confirmed by energy‐dispersive X‐ray spectroscopy (EDS). However, a distinct hollow morphology was observed after etching, and the obtained sample was accompanied by small particles on the outer surface; however, etching did not affect the internal structure of InOF‐1 (Figure 2d). As shown in Figure 2e,f, the elemental maps further confirmed the even distribution of the five elements, and the hollow ZIF‐67 structure was more clearly observed for H‐ZIF‐67‐on‐InOF‐1. More details on ZIF‐67, InOF‐1, and ZIF‐67‐on‐InOF‐1 before and after etching are provided in Figures S16–S18 and Table S3 (Supporting Information), which indicate that acid etching barely affected the microstructures of the different MOF materials.

**Figure 3:**
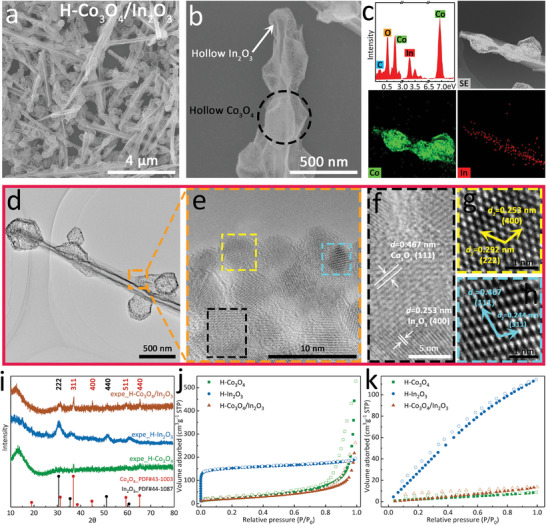
a,b) SEM images and c) EDS profile and element mapping of H‐Co_3_O_4_/In_2_O_3_; d) TEM image, e,f) selected lattice fringes and its Fourier‐transformed ones for g) In_2_O_3_ and h) Co_3_O_4_; i) PXRD patterns, j) N_2_ isotherms, and k) CO_2_ sorption curves of H‐Co_3_O_4_, H‐In_2_O_3_, and H‐Co_3_O_4_/In_2_O_3_.

Direct oxidation was performed to obtain hollow bimetallic oxide nanomaterials, denoted as H‐Co_3_O_4_/In_2_O_3_, with well‐maintained morphological features. The In_2_O_3_ in H‐Co_3_O_4_/In_2_O_3_ showed a wrinkled surface and was closely fused with Co_3_O_4_; however, this phenomenon is not observed for In_2_O_3_ and H‐In_2_O_3_ in **Figure** **3**a,b and Figures S19–S20 (Supporting Information). Meanwhile, a large amount of O species was present on the host In_2_O_3_ and peripheral Co_3_O_4_ with some remaining C and N (Figure 3c and Figure S21, Supporting Information). The TEM images clearly show the exposed nanoparticles in Co_3_O_4_ and the wrinkling and outward extension of the In_2_O_3_ surface. As shown in Figure 3d–h, the two lattice fringes observed at 0.467 and 0.253 nm are attributed to the (111) plane of Co_3_O_4_ and the (400) plane of In_2_O_3_, respectively. Additionally, the diffraction peaks observed at 36.8°, 44.8°, 59.4°, and 65.2° in H‐Co_3_O_4_ and H‐Co_3_O_4_/In_2_O_3_ correspond to the (311), (400), (511), and (440) crystal planes of cobalt oxide (PDF#43‐1003), respectively.^[^ ^33^ ^]^ The (222) and (440) planes of indium oxide (PDF#44‐1087) were also observed for H‐Co_3_O_4_/In_2_O_3_,^[^ ^34^ ^]^ confirming the successful synthesis of the bimetallic oxides (Figure 3i). As shown in Figure 3j and Table S4 (Supporting Information), H‐In_2_O_3_ exhibited type‐I adsorption similar to that of H‐InOF‐1, but with a significant decrease in sorption capacity, and H‐Co_3_O_4_ exhibited a significantly reduced pore volume. Figure S22 (Supporting Information) shows that the pore‐size distribution of H‐Co_3_O_4_/In_2_O_3_ retained the microporous properties of In_2_O_3_, and the addition of Co_3_O_4_ resulted in an increase in mesopores. Furthermore, the CO_2_ isotherms revealed that H‐Co_3_O_4_ had almost no adsorption compared to H‐In_2_O_3_, whereas H‐Co_3_O_4_/In_2_O_3_ exhibited a slightly higher capacity than H‐Co_3_O_4_ (Figure 3k). Thus, the hierarchical nanostructure of the MOF‐on‐MOF‐derived hollow H‐Co_3_O_4_/In_2_O_3_ is considered an excellent heterogeneous oxide catalyst for efficient CO_2_ reduction, where Co_3_O_4_ provides active sites and In_2_O_3_ provides a CO_2_ carrier.

**Figure 4:**
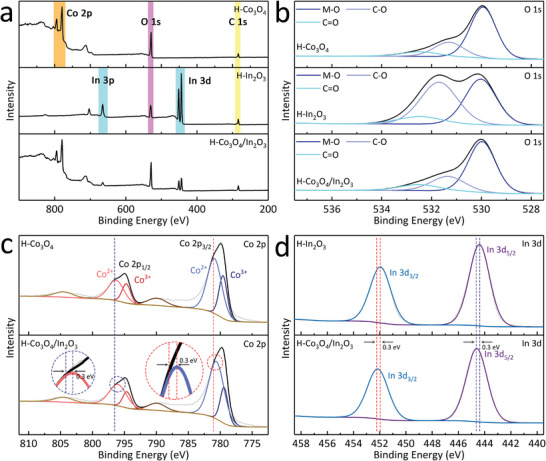
a) Full XPS survey spectra of H‐Co_3_O_4_, H‐In_2_O_3_, and H‐Co_3_O_4_/In_2_O_3_ and their corresponding deconvoluted spectra for b) O 1s, c) Co 2p, and d) In 3d.

X‐ray photoelectron spectroscopy (XPS) was performed to analyze the constituent elements and coordination environment, demonstrating the coexistence of Co, In, and O (**Figure** **4**a). It was clearly observed that the intensity of the O peaks in the full survey spectrum of H‐Co_3_O_4_/In_2_O_3_ was similar to that of H‐Co_3_O_4_. Furthermore, the deconvoluted O 1s peaks observed at 530.0, 531.3, and 532.5 eV are attributed to the M—O, C—O, and C=O bonds, respectively (Figure 4b). As shown in Figure S23 (Supporting Information), the deconvoluted C 1s spectra revealed that the binding energy of the C–C sp^3^ peak in H‐Co_3_O_4_ was positively shifted owing to the larger electronegativity of Co compared to that of In. Similarly, the C–C sp^3^ peak positions in H‐Co_3_O_4_/In_2_O_3_ were also slightly shifted. Weak signals of N were observed in all three samples, which mainly originate from the carbonized linkers in ZIF‐67 or triethylamine during InOF‐1 synthesis (Figure S24, Supporting Information). More importantly, the changes in the coordination environments of Co and In were further investigated. Figure 4c shows two characteristic peaks at ≈781.8/796.3 and 779.5/794.6 eV, corresponding to Co(II) and Co(III), respectively. After oxidation, In atoms form a stable coordination environment with O;^[^ ^35^ ^]^ therefore, two discrete In(III) 3d_5/2_ and 3d_3/2_ peaks were detected (Figure 4d). In addition, there was a slight negative shift in the 2p_1/2_ and 2p_3/2_ peak positions of Co(II) and Co(III) in H‐Co_3_O_4_/In_2_O_3_ compared to those in H‐Co_3_O_4_, whereas the binding energy of In(III) shifted in the positive direction compared to that of H‐In_2_O_3_.^[^ ^36^ ^]^ Co in H‐Co_3_O_4_/In_2_O_3_ easily combines with O, which shifts the electrons on Co toward O, while In strengthens the binding ability, resulting in a slight increase in the binding energy. More detailed XPS data is presented in Table S5 (Supporting Information).

**Figure 5:**
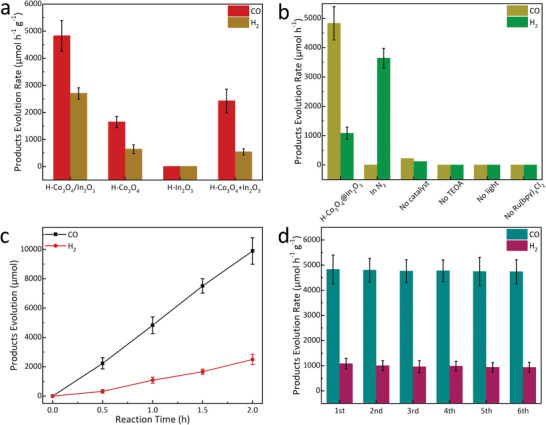
a) Photocatalytic activity of H‐Co_3_O_4_/In_2_O_3_ and the control samples; b–d) Control experiments under different test conditions, time‐product yield plots, and cycling stability tests.

The photocatalytic activity was evaluated in a mixed solvent containing triethanolamine (TEOA) and [Ru(bpy)_3_]Cl_2_ as the sacrificial agent and photosensitizer, respectively. For better comparison, three control samples were also prepared under the same conditions: H‐Co_3_O_4_, H‐In_2_O_3_, and physically mixed H‐Co_3_O_4_+In_2_O_3_. Among them, H‐Co_3_O_4_/In_2_O_3_ exhibited the best activity with a high CO production rate of 4828 ± 570 µmol h^−1^ g^−1^, which was higher than those of H‐Co_3_O_4_ (1644 ± 210 µmol h^−1^ g^−1^) and H‐Co_3_O_4_+In_2_O_3_ (2420 ± 444 µmol h^−1^ g^−1^). As shown in **Figure** **5**a, H‐In_2_O_3_ exhibited almost no photocatalytic activity, indicating that the active sites in H‐Co_3_O_4_/In_2_O_3_ are mainly provided by Co_3_O_4_, because H‐Co_3_O_4_/In_2_O_3_ exhibited a high selectivity of 80%,^[^ ^37^, ^38^ ^]^ and the apparent quantum efficiency for CO generation at 450 nm was calculated to be 0.59%. Other control experiments were performed to investigate the effects of internal conditions and external factors on the CO_2_RR, including the carbon source, photocatalyst, sacrificial agent, photosensitizer, and light source (Figure 5b). In addition to the activity and selectivity, the optimal H‐Co_3_O_4_/In_2_O_3_ exhibited a durable CO_2_ photoreduction performance for over 2 h, and no substantial deactivation was observed after six recycling tests (Figure 5c,d).^[^ ^39^, ^40^, ^41^, ^42^ ^]^

**Figure 6:**
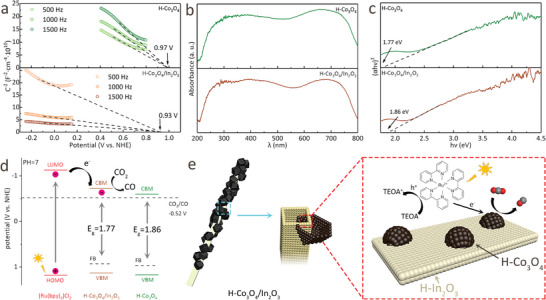
a) Mott–Schottky plots, b) UV–vis–NIR DRS spectra, and c) Tauc plots for H‐Co_3_O_4_ and H‐Co_3_O_4_/In_2_O_3_; d) Band alignments of [Ru(bpy)_3_]Cl_2_, H‐Co_3_O_4_, and H‐Co_3_O_4_/In_2_O_3_; e) Proposed catalytic mechanism for the photoreduction of CO_2_ to CO over H‐Co_3_O_4_/In_2_O_3_.

The Mott–Schottky plots of H‐Co_3_O_4_ and H‐Co_3_O_4_/In_2_O_3_ show negative slopes, indicating that the materials are p‐type semiconductors, while the plot for H‐Co_3_O_4_/In_2_O_3_ confirms that H‐Co_3_O_4_ plays a dominant role in H‐Co_3_O_4_/In_2_O_3_ (**Figure** **6**a).^[^ ^43^, ^44^ ^]^ Meanwhile, the flat band potentials (*E*_FB_) of H‐Co_3_O_4_ and H‐Co_3_O_4_/In_2_O_3_ were calculated to be 0.97 and 0.93 V versus a normal hydrogen electrode (NHE), respectively. Generally, it is recognized that the valence band (*E*_VB_) of p‐type semiconductors is 0.2 V more positive than that of *E*_FB_.^[^ ^45^, ^46^ ^]^ Therefore, the *E*_VB_ values of H‐Co_3_O_4_ and H‐Co_3_O_4_/In_2_O_3_ were calculated to be 1.17 and 1.13 V (vs NHE), respectively. According to the band gap data in Figure 6b,c, the *E*_g_ values of H‐Co_3_O_4_ and H‐Co_3_O_4_/In_2_O_3_ were 1.77 and 1.86 eV, respectively. Thus, the *E*_CB_ values of H‐Co_3_O_4_ and H‐Co_3_O_4_/In_2_O_3_ were calculated to be −0.60 and −0.73 V (vs NHE), respectively. It is demonstrated that the lowest unoccupied molecular orbital (LUMO) and highest occupied molecular orbital (HOMO) energy levels of [Ru(bpy)_3_]Cl_2_ were −1.13 and 1.18 V (vs NHE), respectively. The calculated band structures of the samples are shown in Figure 6d and Table S6 (Supporting Information). Based on the above‐mentioned results, the band structures of H‐Co_3_O_4_ and H‐Co_3_O_4_/In_2_O_3_ correspond well with that of [Ru(bpy)_3_]Cl_2_, and the photogenerated electrons are transferred from the photosensitizer to the synthesized photocatalyst.^[^ ^47^, ^48^ ^]^ In contrast, the conduction band (CB) positions of H‐Co_3_O_4_ and H‐Co_3_O_4_/In_2_O_3_ were more negative than the reduction potential of CO_2_ to CO (−0.52 V vs NHE). However, the more negative CB position of H‐Co_3_O_4_/In_2_O_3_ endows it with a stronger CO_2_ conversion reduction potential than H‐Co_3_O_4_. Finally, a feasible mechanism for CO_2_ photoreduction over H‐Co_3_O_4_/In_2_O_3_ is proposed (Figure 6e), in which the photosensitizer is first excited and then quenched by TEOA to form the reduced state. Subsequently, photoinduced electron transfer occurs from the LUMO of [Ru(bpy)_3_]Cl_2_ to the CB of H‐Co_3_O_4_/In_2_O_3_. Finally, the adsorbed CO_2_ is rapidly reduced to CO, which is desorbed from the photocatalyst surface.^[^ ^49^, ^50^ ^]^

## 3 Conclusion

In summary, this study demonstrated the successful fabrication of a unique hollow heterostructure, H‐ZIF‐67‐on‐InOF‐1, which was further treated to generate bimetallic oxide nanomaterials. The obtained MOF‐on‐MOF‐derived hollow H‐Co_3_O_4_/In_2_O_3_ exhibited numerous heterogeneous interfaces, where the Co‐based oxide species acted as active sites, whereas the In‐based oxide species served as CO_2_ carriers for efficient CO_2_ reduction. The optimal H‐Co_3_O_4_/In_2_O_3_ exhibited a satisfactory photocatalysis performance with a highest CO production rate of 4828 ± 570 µmol h^−1^ g^−1^. Moreover, it showed better photo‐responsiveness, catalytic activity, and robust photo‐stability than the control samples. Theoretically, this composite results in a rapid electron transfer under light irradiation, indicating a strong synergistic effect on the CO_2_RR by integrating H‐Co_3_O_4_ and H‐In_2_O_3_. Finally, our study can be extended to the rational design and structural control of MOF precursors and their derivatives as efficient and durable catalysts in the context of pollutant degradation, water splitting, fuel cells and other applications.

## Conflict of Interest

The authors declare no conflict of interest.

## Author Contributions

J.J.Q. and T.T.L. provided ideas for this study and designed the relevant experiments. J.J.Q. and C.H. synthesized and characterized the samples. J.J.Q., T.T.L., and C.H. analyzed and summarized the experimental results and wrote the manuscript. All other authors offered great help during submission.

## Supporting information

Supporting Information

## Data Availability

The data that support the findings of this study are available from the corresponding author upon reasonable request.
