# Supplementary material for: MOF‐on‐MOF‐Derived Hollow Co3O4/In2O3 Nanostructure for Efficient Photocatalytic CO2 Reduction
Source: Adv Sci (Weinh). 2023 Apr 21;10(19):2300797. doi: 10.1002/advs.202300797 (PMC10323637; doi:10.1002/advs.202300797)
Supplement: Supplementary file 1 — Supporting Information [file ADVS-10-2300797-s001.pdf]

ADVANCED SCIENCE
Open Access

Supporting Information

for *Adv. Sci.*, DOI 10.1002/advs.202300797

MOF-on-MOF-Derived Hollow Co_3O_4/In_2O_3 Nanostructure for Efficient Photocatalytic CO_2 Reduction

Cheng Han, Xiaodeng Zhang, Shengsheng Huang, Yue Hu, Zhi Yang, Ting-Ting Li, Qipeng Li* and Jinjie Qian**

MOF-on-MOF Derived Hollow Co_3O_4/In_2O_3 Nanostructure for Efficient Photocatalytic CO_2 Reduction

Cheng Han,[a] Xiaodeng Zhang,[a] Shengsheng Huang,[c] Yue Hu,[a] Zhi Yang,[a] Ting-Ting Li,[c] Qipeng Li[*b] and Jinjie Qian[*a]

[a]Key Laboratory of Carbon Materials of Zhejiang Province, College of Chemistry and Materials Engineering, Wenzhou University, Wenzhou 325000, P. R. China.

[b]College of Chemistry and Chemical Engineering, Zhaotong University, Zhaotong, Yunnan, P. R. China, 657000.

[c]School of Materials Science and Chemical Engineering, Ningbo University, Ningbo 315211, P. R. China.

*Corresponding author

E-mail: liqipeng10@mails.ucas.ac.cn; jinjieqian@wzu.edu.cn.

Experiment

Synthesis of InOF-1

A mixed solution of $In(NO_3)_3$ xH_2O (0.10 mmol, 30 mg), H_4BPTC (0.05 mmol, 15 mg) and CTAB (0.01 mmol, 5 mg) with DMF (3 mL) and H_2O (3 mL) is prepared at 35 mL pressure-resistant tube, additionally add 0.1 mL HNO_3 and 0.1 mL TEA, and sonicate until no obvious particles exist at the bottom of the solution. Then the solution is heated at 140 °C for 30 min and cooled to room temperature. After several washes with DMF and EtOH, InOF-1 nanorods are collected at a centrifuge speed of 10000 rpm and finally dried in vacuum at 85 °C. The yield of InOF-1 is about 57% based on the initial organic formulation.

Synthesis of ZIF-67

$Co(NO_3)_2{\cdot}6H_2O$ (0.34 mmol, 99.6 mg) is mixed with 5 mL MeOH and stirred thoroughly for 1 h to form solution A; HMeIm (1.86 mmol, 153 mg) is dissolved in 5 mL MeOH to form solution B.Then the mixture of solutions A and B is heated and stirred at 70 °C for 20 min, centrifuged at 9500 rpm for 3 min to collect the precipitate and finally washed with EtOH solvent for 3 times and dried under vacuum at 85 °C overnight to obtain dodecahedral ZIF-67 particles.

Synthesis of ZIF-67-on-InOF-1

10 mg InOF-1 and 35 mg PVP (58000) are well dispersed in 5 mL MeOH, then $Co(NO_3)_2{\cdot}6H_2O$ (0.34 mmol, 99.6 mg) is dissolved in the above solution and stirred for 1 hour to form solution A; HMeIm (1.86 mmol, 153 mg) is dissolved in 5 mL

MeOH to form solution B. Then the mixture of solutions A and B is heated and stirred at 70 °C for 20 min, centrifuged at 9500 rpm for 3 min to collect the precipitate and finally washed with EtOH solvent for 3 times and dried under vacuum at 85 °C to obtain ZIF-67-on-InOF-1.

Acid-Etching of ZIF-67, InOF-1 and ZIF-67-on-InOF-1

First, 50 mg of cyanuric acid (CA) is dissolved in 50 ml of EtOH and heated at 60 °C for 30 min to completely dissolve CA. Subsequently, 50 mg of the sample is added to the solution and ultra-sonicated for 5 min to make it uniformly dispersed. Finally, heat this solution at 60 °C for 1 day. The precipitate is collected by centrifugation at 9500 rpm for 3 min, washed with EtOH three times and dried in vacuum at 85 °C overnight to obtain H-ZIF-67, H-InOF-1 and H-ZIF-67-on-InOF-1.

Synthesis of H-Co_3O_4, H-In_2O_3 and H-Co_3O_4/In_2O_3

The synthesized H-ZIF-67, H-InOF-1 and H-ZIF-67-on-InOF-1 are transferred into a muffle furnace and oxidized in air. The specific scheme is as follows: the sample is heated from room temperature to 350 °C at a rate of 10 °C min^{-1}, kept at 350 °C for 2 h and then naturally cooled to room temperature to obtain H-Co_3O_4, H-In_2O_3 and H-Co_3O_4/In_2O_3.

Photocatalytic experiments:

The photocatalytic experiments are performed in a 50 mL gas-closed quartz reactor. The photocatalytic system containing $[Ru(bpy)_3]Cl_2·6H_2O$ (10 mg), photocatalyst (5 mg), and solvent [10 mL, MeCN/TEOA/H_2O = 3:1:1 (vol/vol))]. Before photoirradiation, the sealed reactor is vacuum degassed, and then the high-purity CO_2 is filled into the reactor for several times to ensure a CO_2 pressure of 1 bar. During photocatalytic experiment, the system is stirred with a magnetic stirrer and irradiates under a 300 W Xenon lamp (Beijing Ceaulight Company) with a 420 nm cutoff filter. The optical density of the light source is 220 mW cm^{-2}, as determined by an optical power meter, and the illuminated area is 15.20 cm^2. After the experiment, the possible gas products (*e.g.,* CO, H_2) are detected by gas chromatography. Liquid products (*e.g.,* HCOOH, CH_3OH) are analyzed by ^1H NMR spectroscopy. For the recycling experiments, the test time for each cycle is 3 h, after the first each cycle, the used photocatalyst is washed by MeCN and H_2O, re-collected by centrifugation, and continuously used for next cycle.

The determination of apparent quantum efficiency:

The estimation of apparent quantum efficiency (AQE) is obtained by a monochromatic light irradiation CO_2 photoreduction test. The AQE value at 450 nm for CO or H_2 generation is determined by a band pass filter on a same photocatalytic setup. The lamp intensity at 450 nm is measured to be 27.8 mW cm^{-2}, and the illuminated area is 15.20 cm^2. The corresponding calculation equation is shown as follows:

$$AQE(CO/H_2) = \frac{number\, of\ generater CO/H_2 \times 2}{number\, of\ incident\, photons} \times 100\% = \frac{2 \times n_{CO/H_2)} \times N_A \times hc}{PSt\lambda} \times 100\%$$

where n_{CO} and n_{H2}: the generated mole number (mol) of CO and H_2, respectively; N_A: Avogadro's constant (6.02×10^{23} mol^{-1}); *h:* Planck's constant (6.626×10^{-34} J s^{-1}); *P:* the power intensity of the Xe lamp at a specific wavelength (mW cm^{-2}); *S:* the effective illumination area; *t:* irradiation time (3600 s); *λ:* monochromatic light wavelength (nm); and *c:* the speed of light (3×10^{8} m s^{-1}).

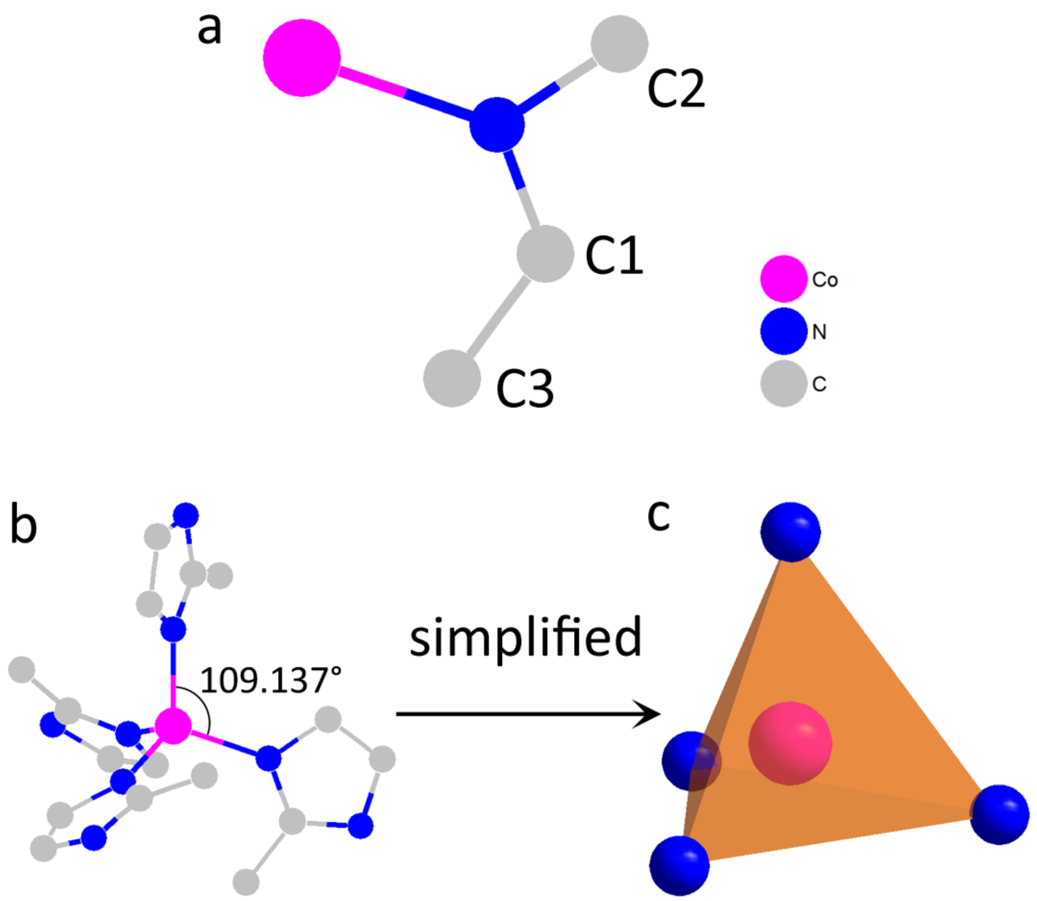

Figure S1. The (a) AU and (b) SBU of ZIF-67, it can be observed that the SBU presents 4-coordinated Co, and the angle between each Co and N is 109.137°, simplified into a regular tetrahedral SBU.

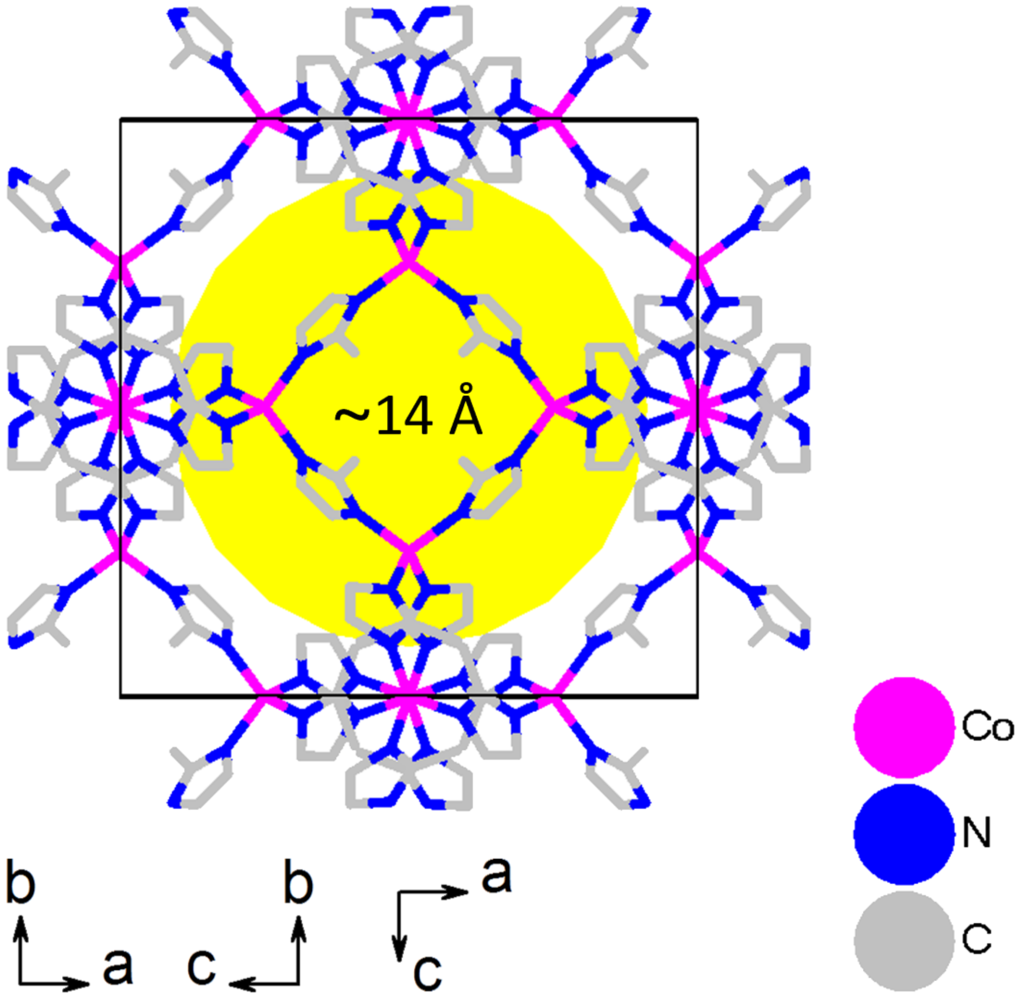

Figure S2. A 0-dimensional cage in a ZIF-67 unit cell that can pack guest molecules with a diameter of about 14 Å. The same structure can be observed along the *a, b, c* axes.

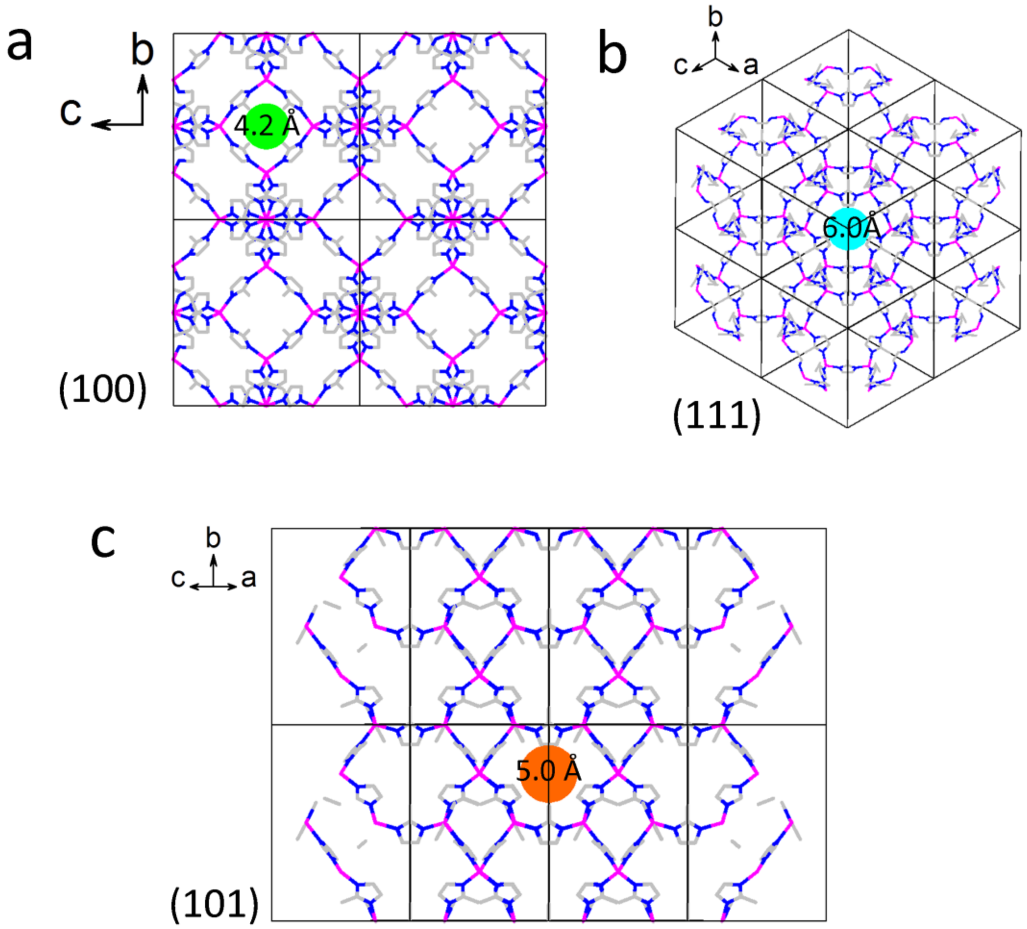

Figure S3. Along different directions, three kinds of 1-dimensional tubular channels can be observed in ZIF-67, with diameters of about 4.2 Å, 6.0 Å and 5.0 Å.

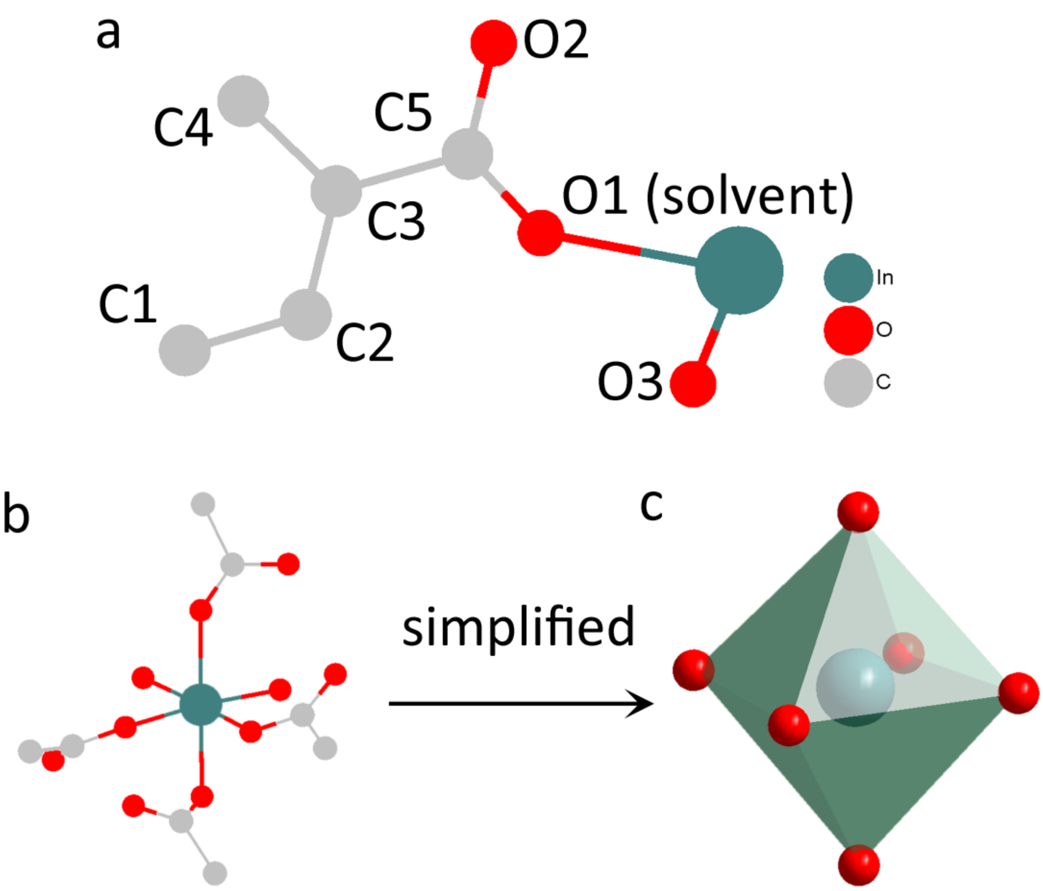

Figure S4. The (a) AU and (b) SBU of InOF-1, it can be observed that the SBU presents 6-coordinated O, where two O atoms come from the solvent, simplified into an octahedron SBU.

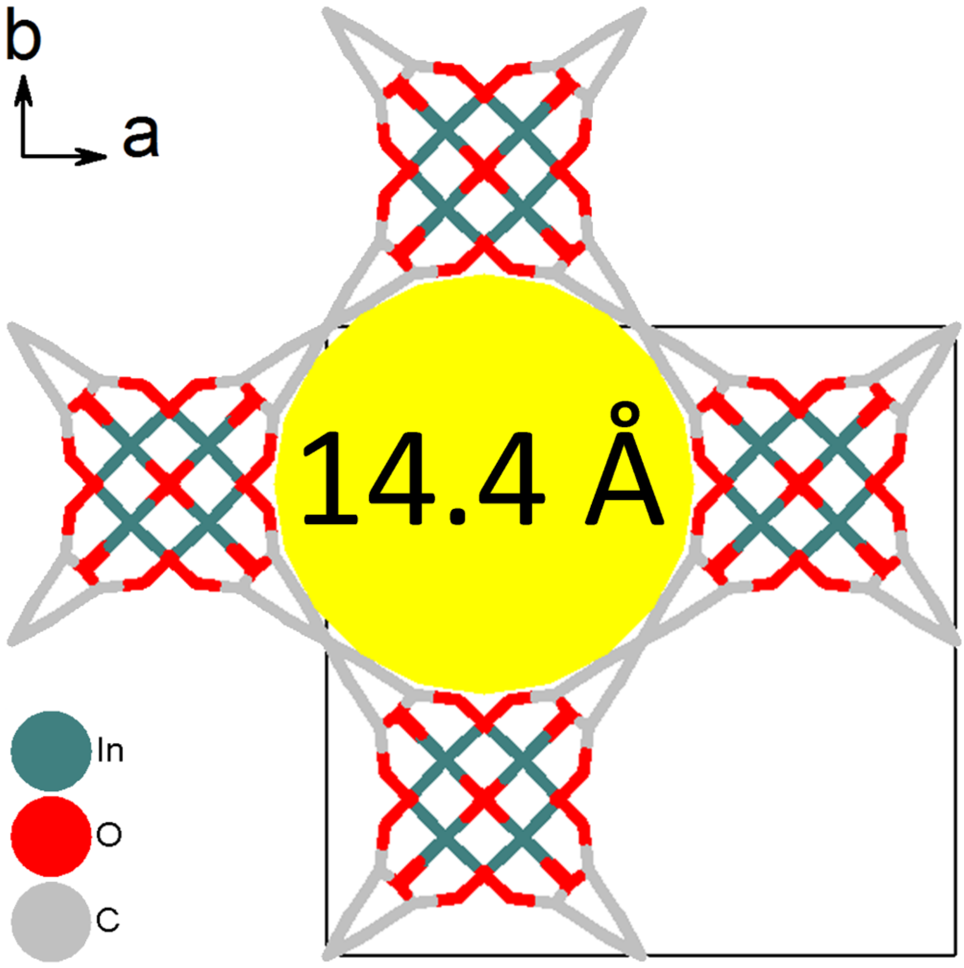

Figure S5. A 1-dimensional channel in InOF-1 that can accommodate guest molecules with an average diameter of 14.4 Å.

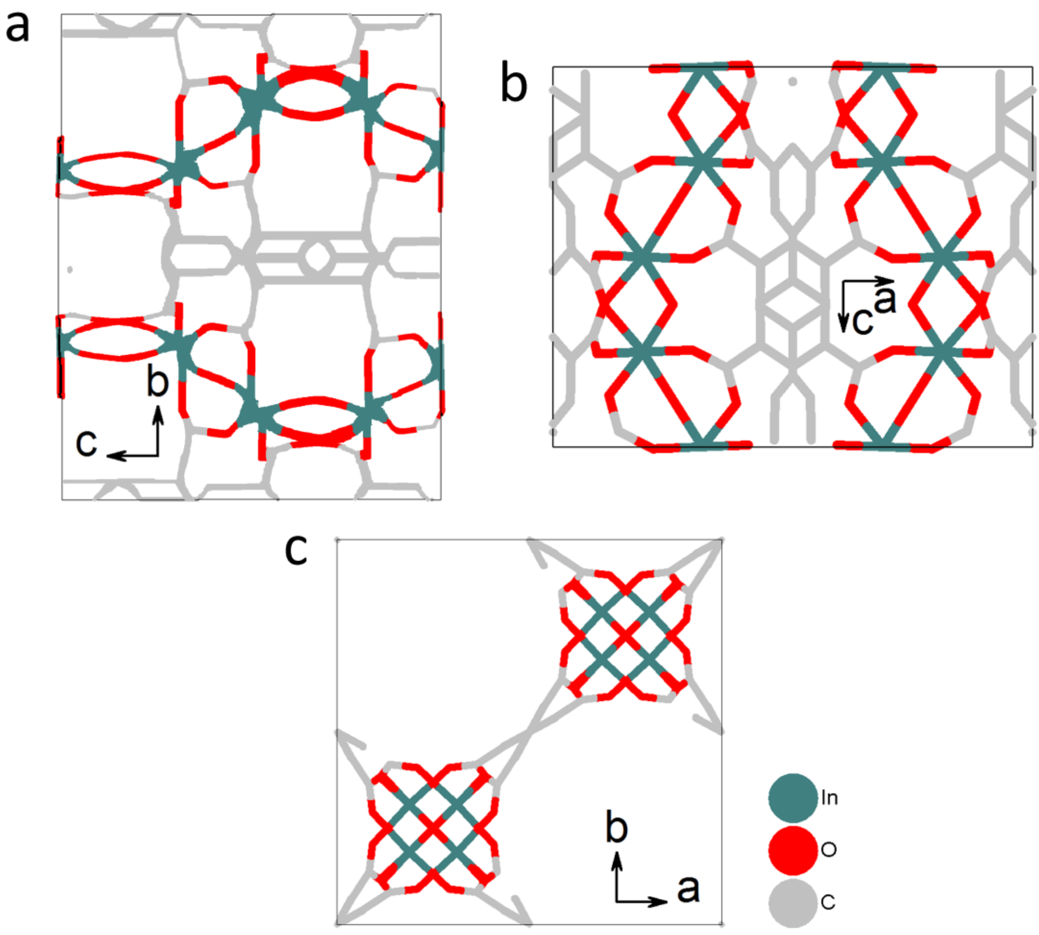

Figure S6. Structure of InOF-1 observed along different axes.

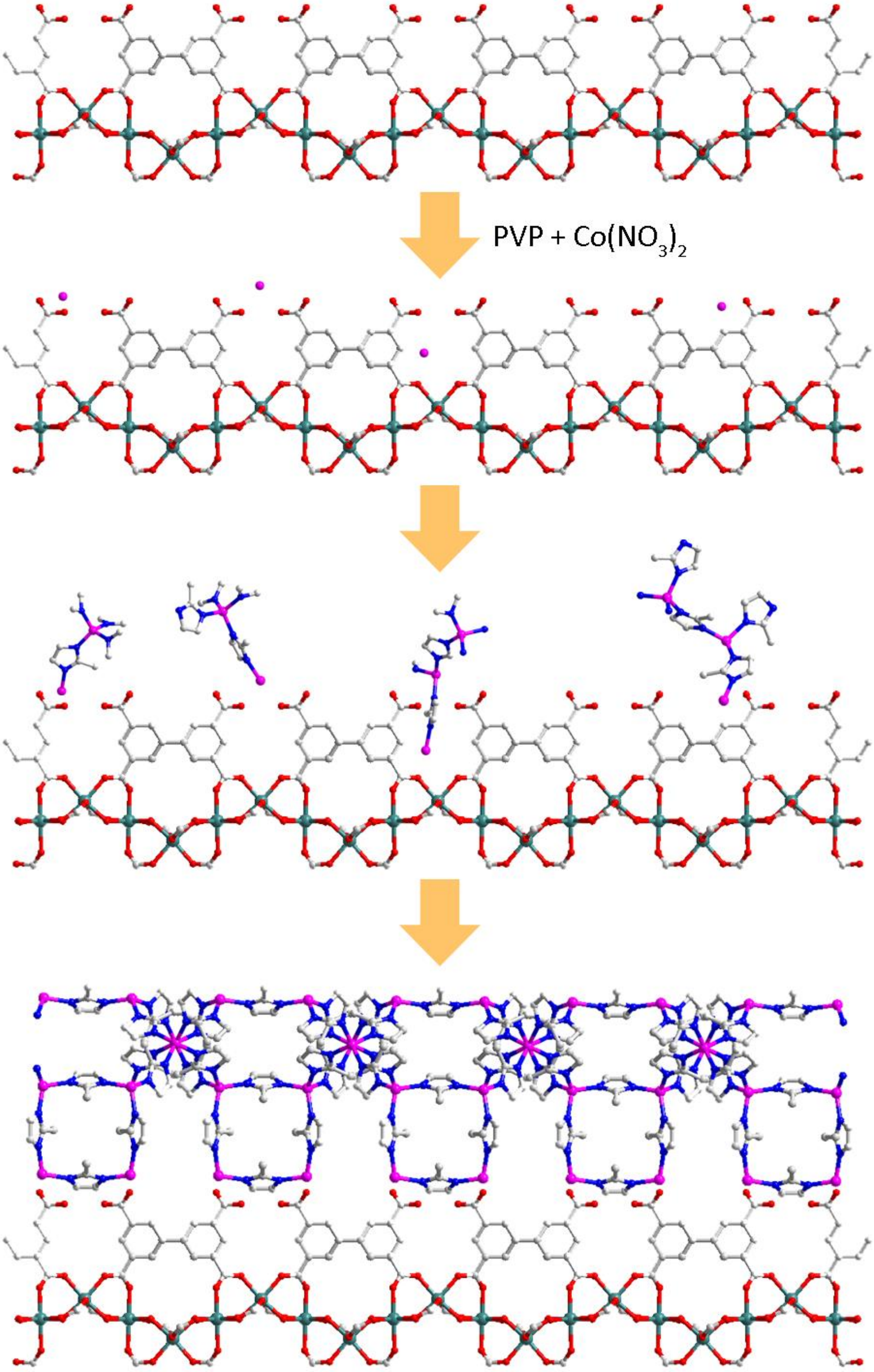

Figure S7. The higher electronegativity of O atoms in InOF-1 can strongly attract Co(II) ions, so it can be fully dispersed between the pores and surfaces of InOF-1 with polyvinylpyrrolidone to facilitate the secondary ZIF-67 growth.

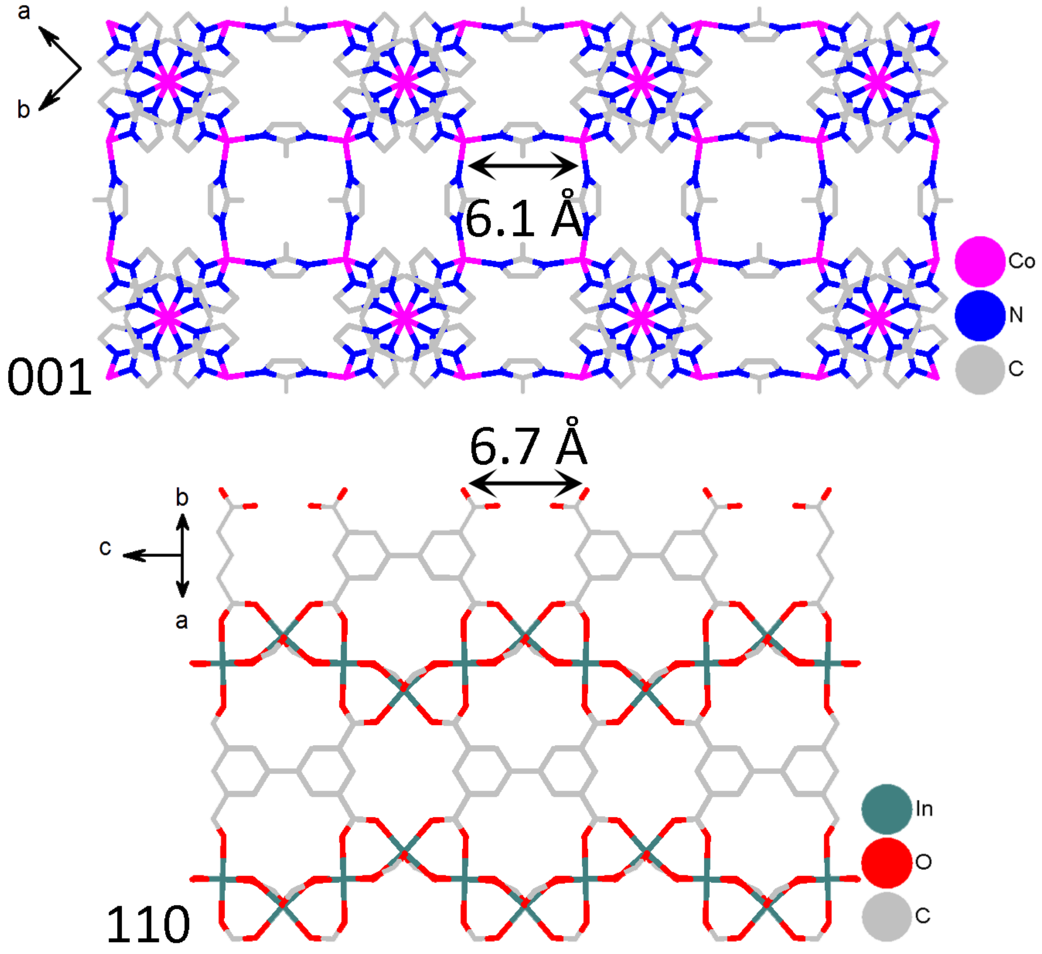

Figure S8. The distances between the two SBUs of ZIF-67 and two adjacent ligands in InOF-1 are almost identical, which can ensure the secondary growth of ZIF-67 on InOF-1.

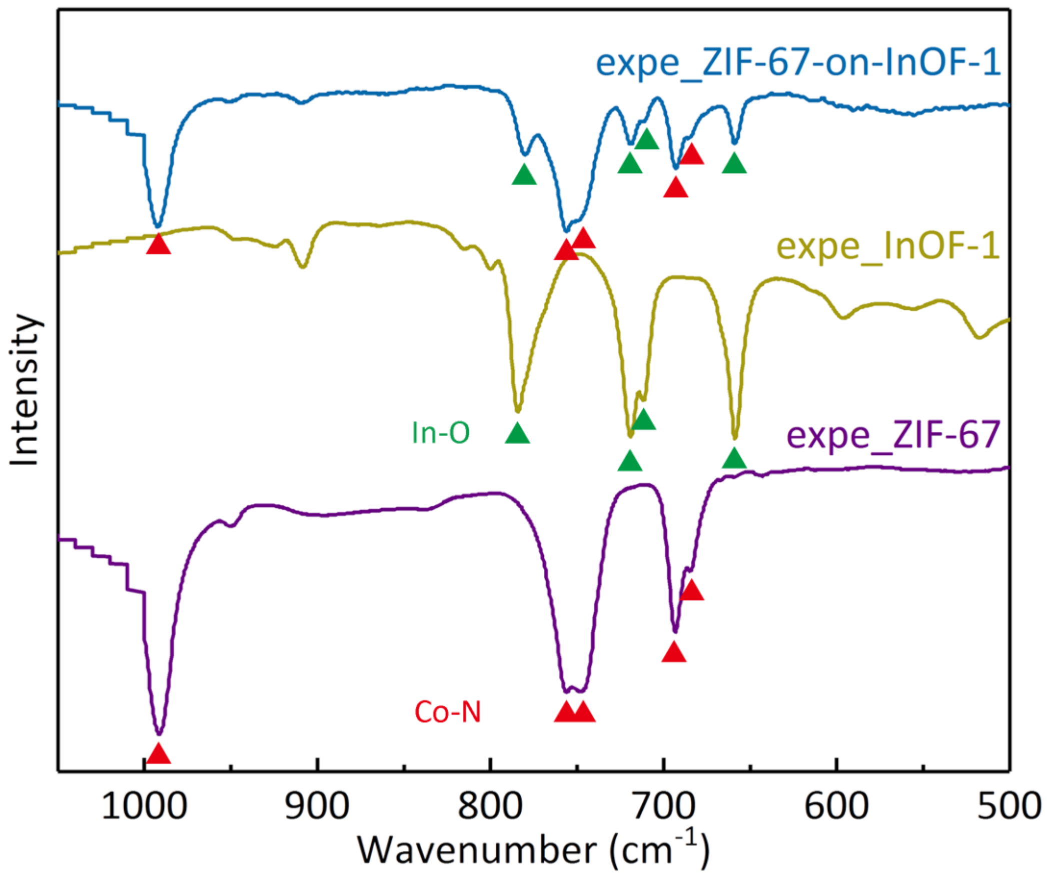

Figure S9. In the FT-IR spectra, it can be clearly observed that ZIF-67-on-InOF-1 contains two characteristic sets of peaks (500-1050 cm^{-1}) corresponding to the Co-N bond in ZIF-67 and the In-O bond in InOF-1 respectively.

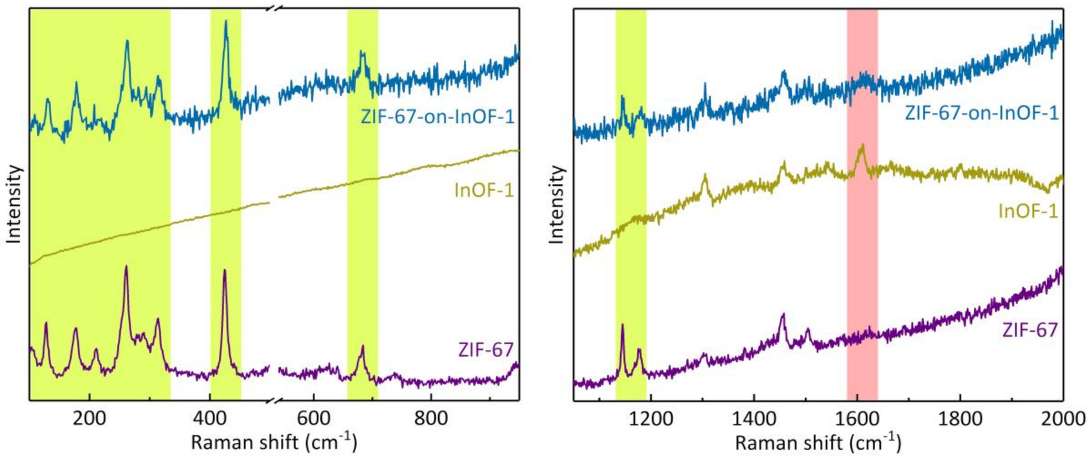

Figure S10. For Raman spectra from 100 to 2000 cm^{-1}, distinct ZIF-67 peaks (0-330 cm^{-1}, 432 cm^{-1}, 780 cm^{-1} and 1050-1196 cm^{-1}) and InOF-1 peaks (1600 cm^{-1}) in the composite are observed. A break point near 520 cm^{-1} is to prevent the influence of the Si substrate peak.

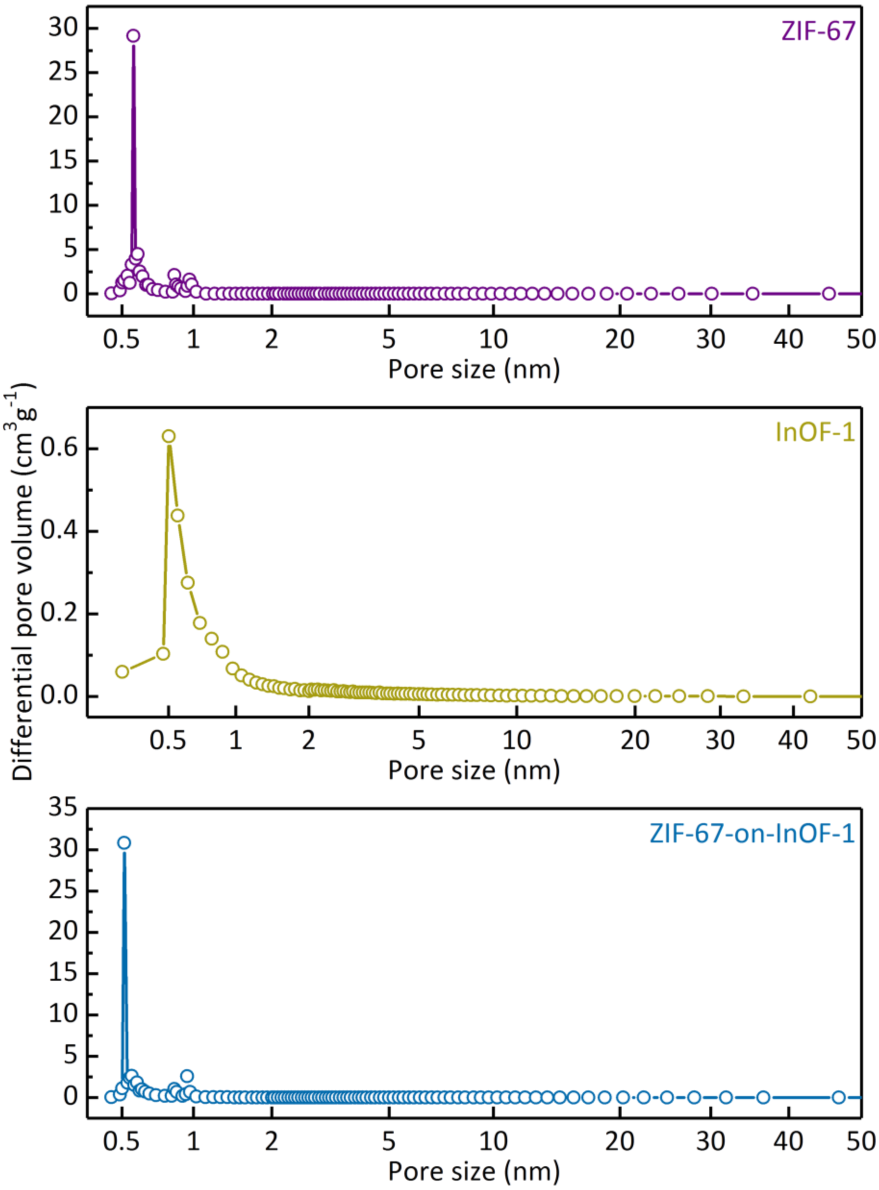

Figure S11. The pore size distributions of ZIF-67, InOF-1 and ZIF-67-on-InOF-1, all three materials have micropore characteristics..

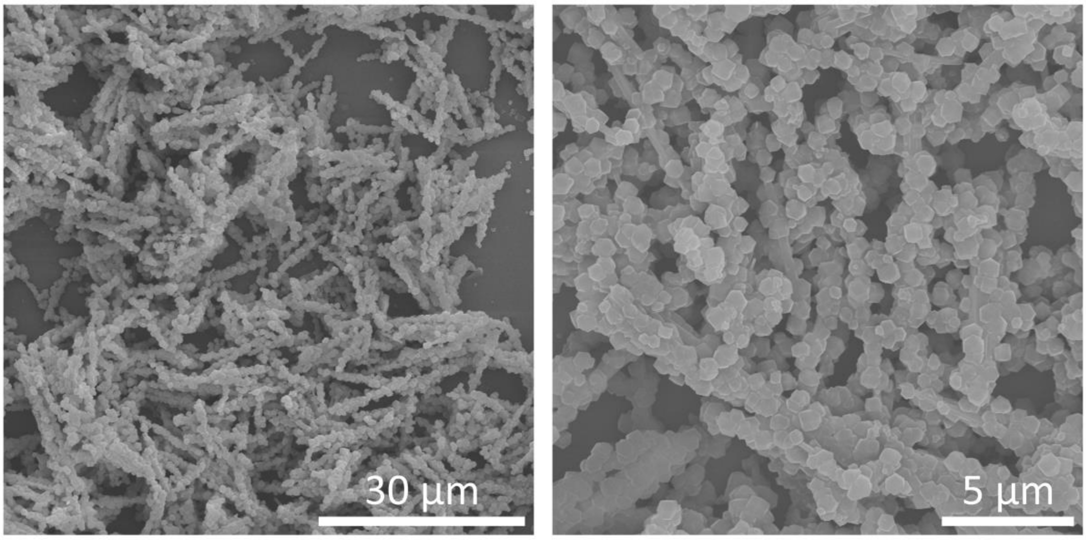

Figure S12. SEM images of ZIF-67-on-InOF-1 to show the surface of the rod-shaped InOF-1 fully encapsulated by ZIF-67.

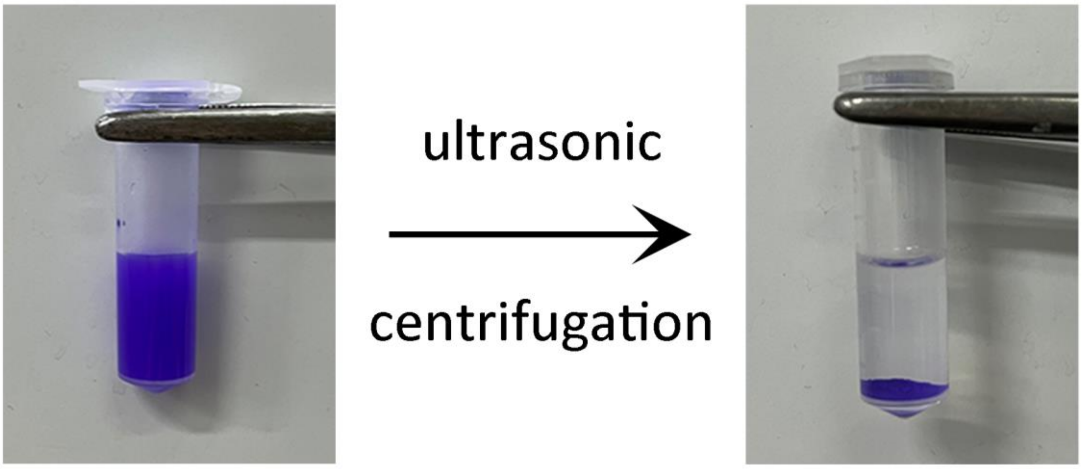

Figure S13. ZIF-67-on-InOF-1 sample and supernatant color change after sonication in ethanol and centrifugation.

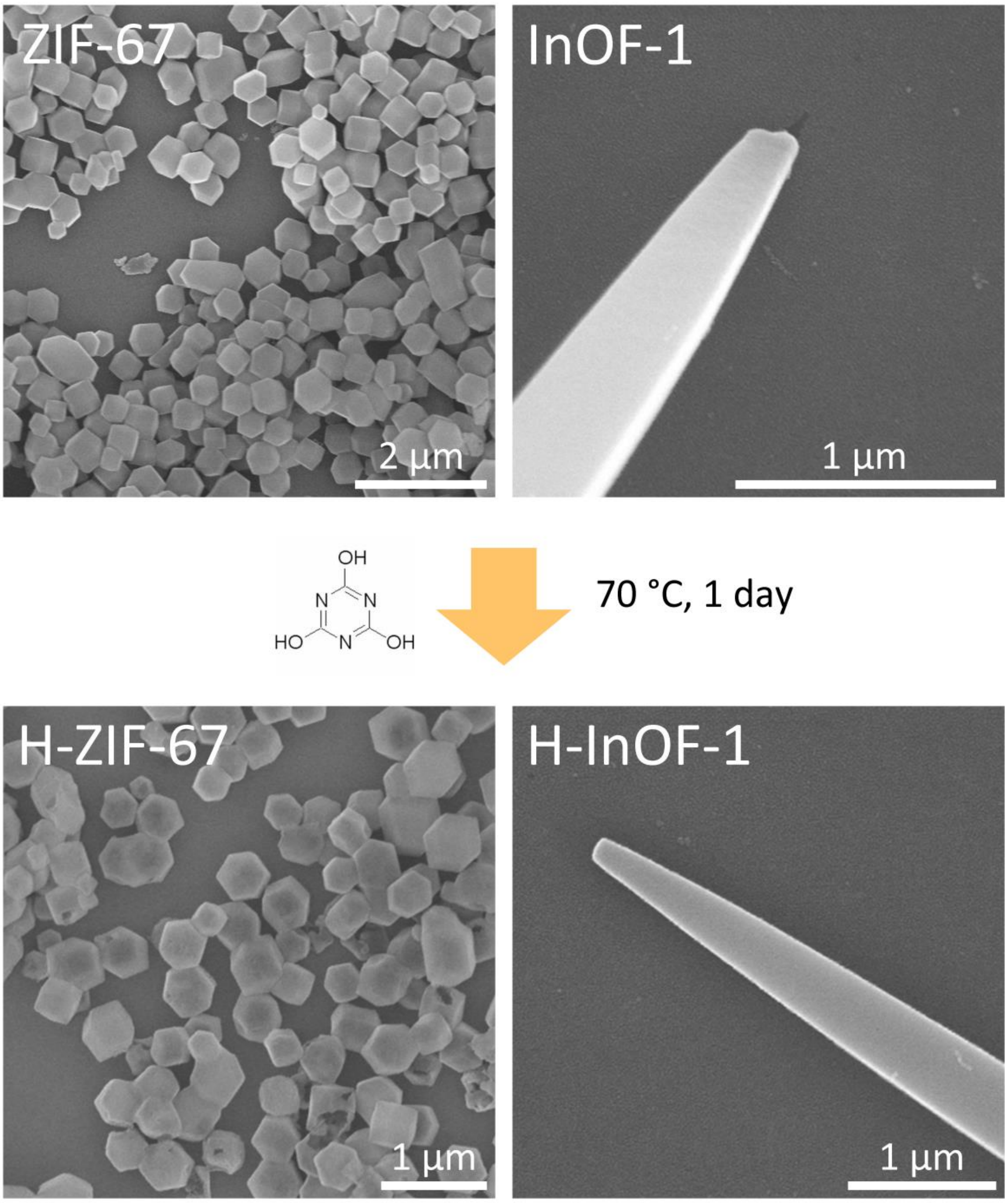

Figure S14. The SEM images of H-ZIF-67 and H-InOF-1 after cyanuric acid (CA) etching, H-ZIF-67 shows obvious hollow properties, while H-InOF-1 hardly changes after acid etching.

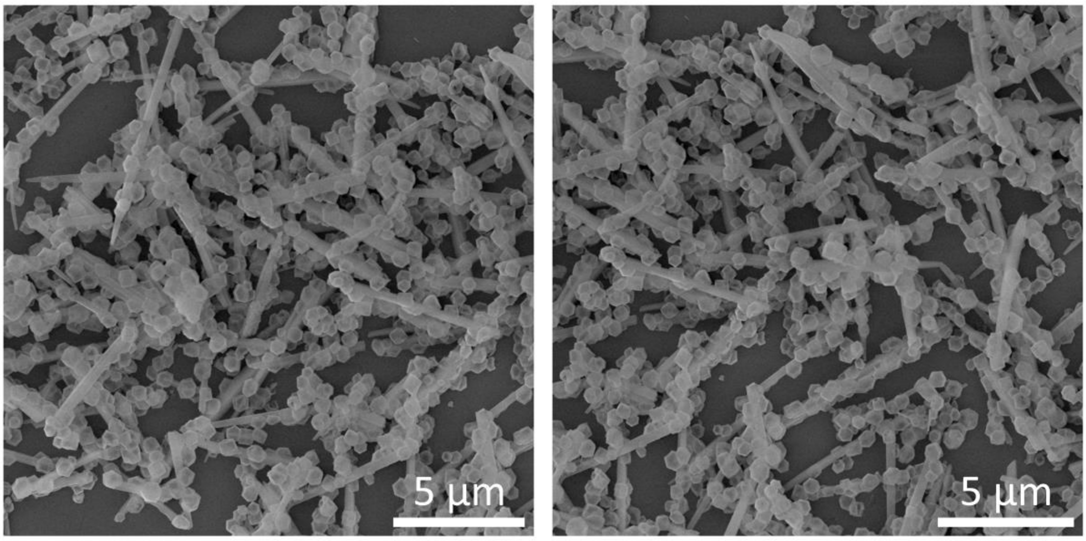

Figure S15. The SEM images of H-ZIF-67-on-InOF-1 after CA etching, the ZIF-67 supported on the InOF-1 surface has formed a hollow morphology.

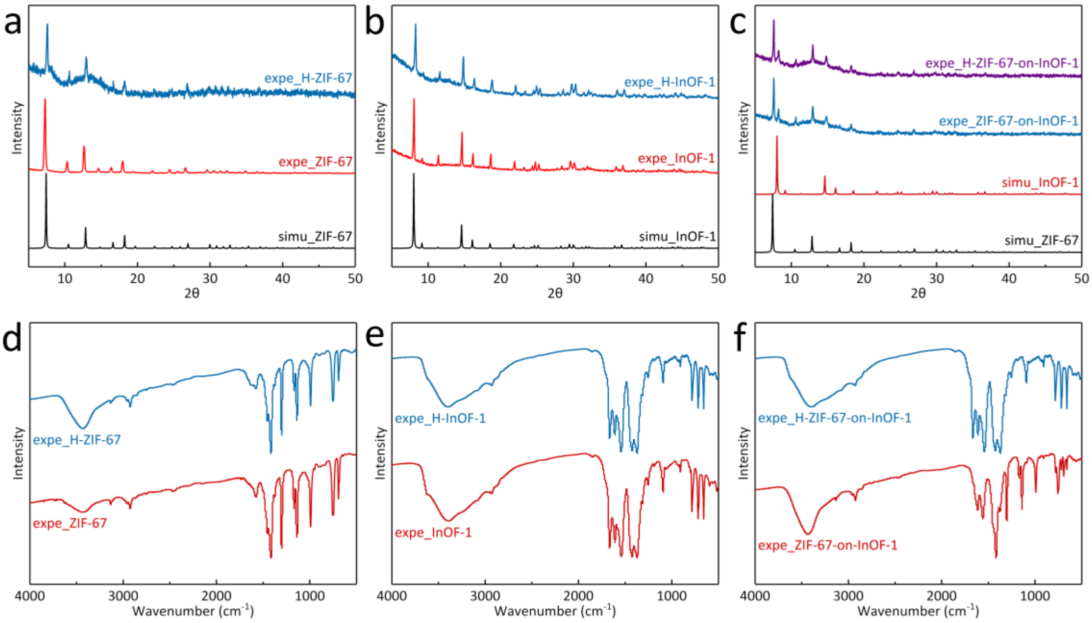

Figure S16. (a-c) PXRD and (d-f) FT-IR of ZIF-67, InOF-1 and ZIF-67-on-InOF-1 before and after etching by CA. The positions of all signal peaks do not change, which prove that the acid etching process has no effect on the properties and phases of the materials, and the etched materials retain their original characteristics.

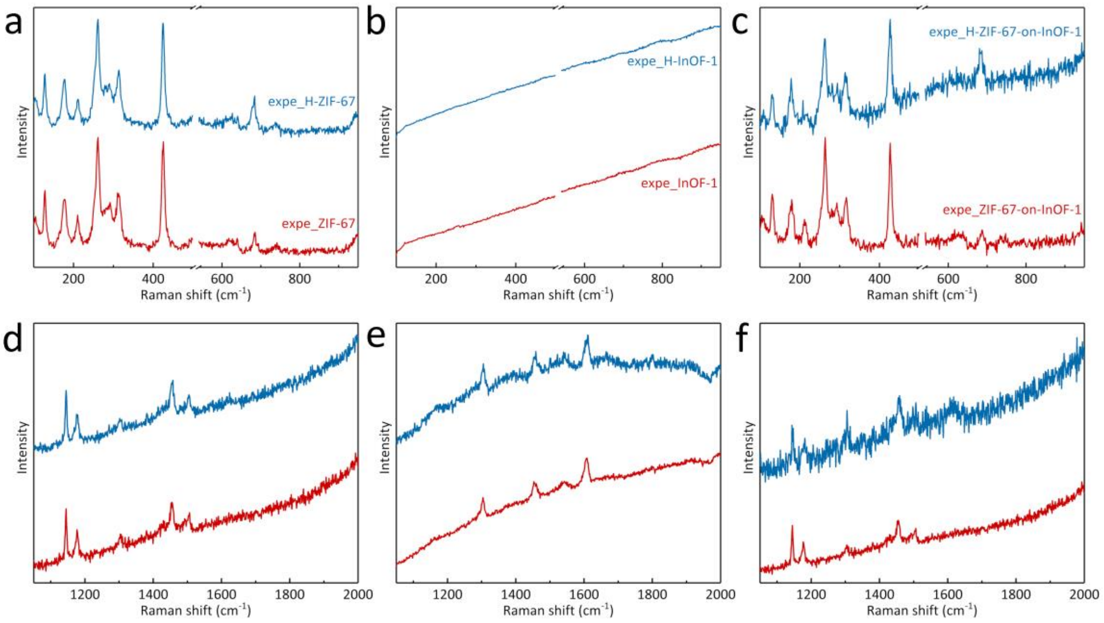

Figure S17. Raman spectra of (a/d) ZIF-67, (b/e) InOF-1 and (c/f) ZIF-67-on-InOF-1 before/after etching by CA.

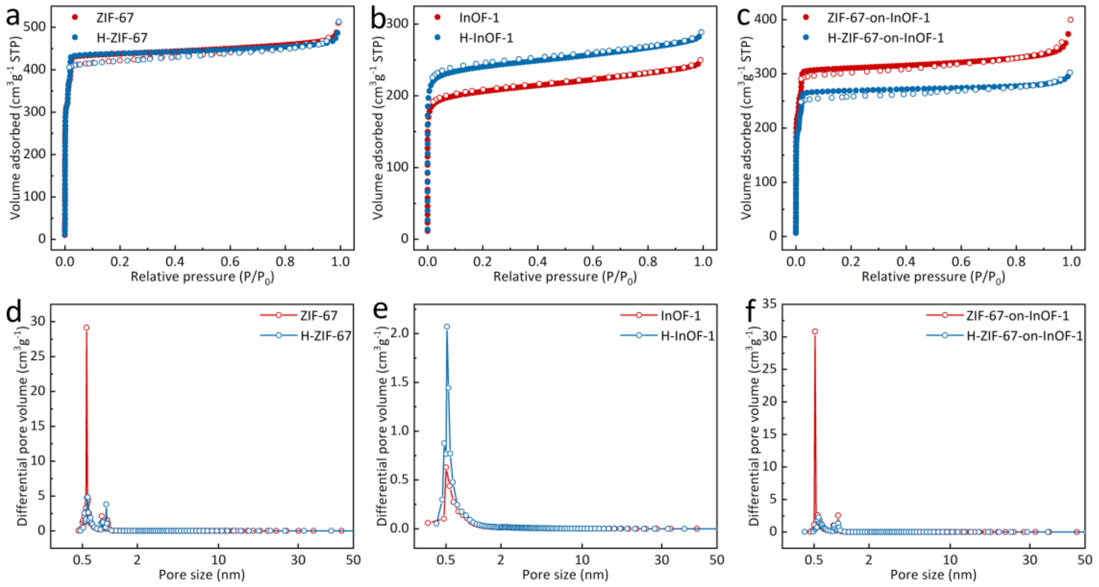

Figure S18. Nitrogen isotherms/PSD curves of (a/d) ZIF-67, (b/e) InOF-1 and (c/f) ZIF-67-on-InOF-1 before and after etching by CA.

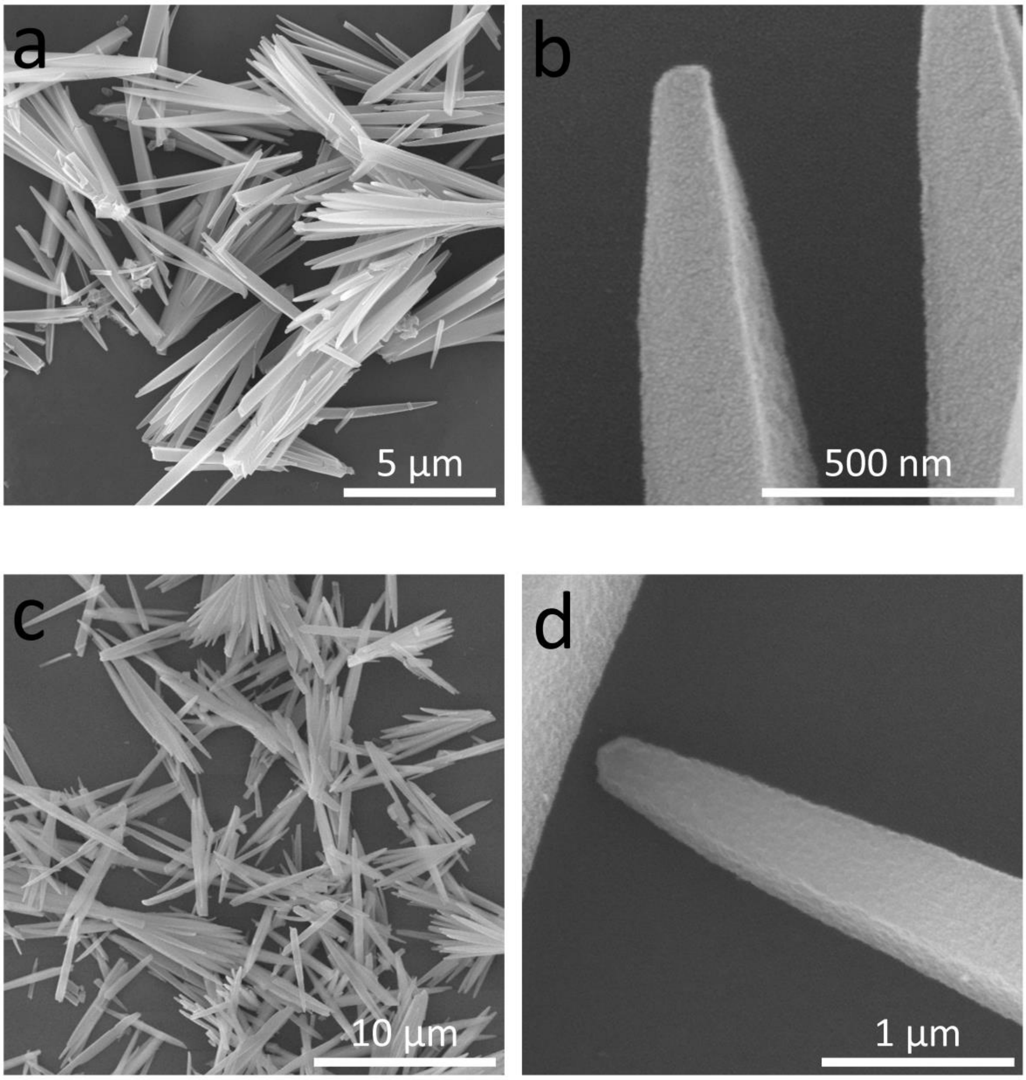

.

Figure S19. SEM images of (a, b) In_2O_3 and (c, d) H-In_2O_3.

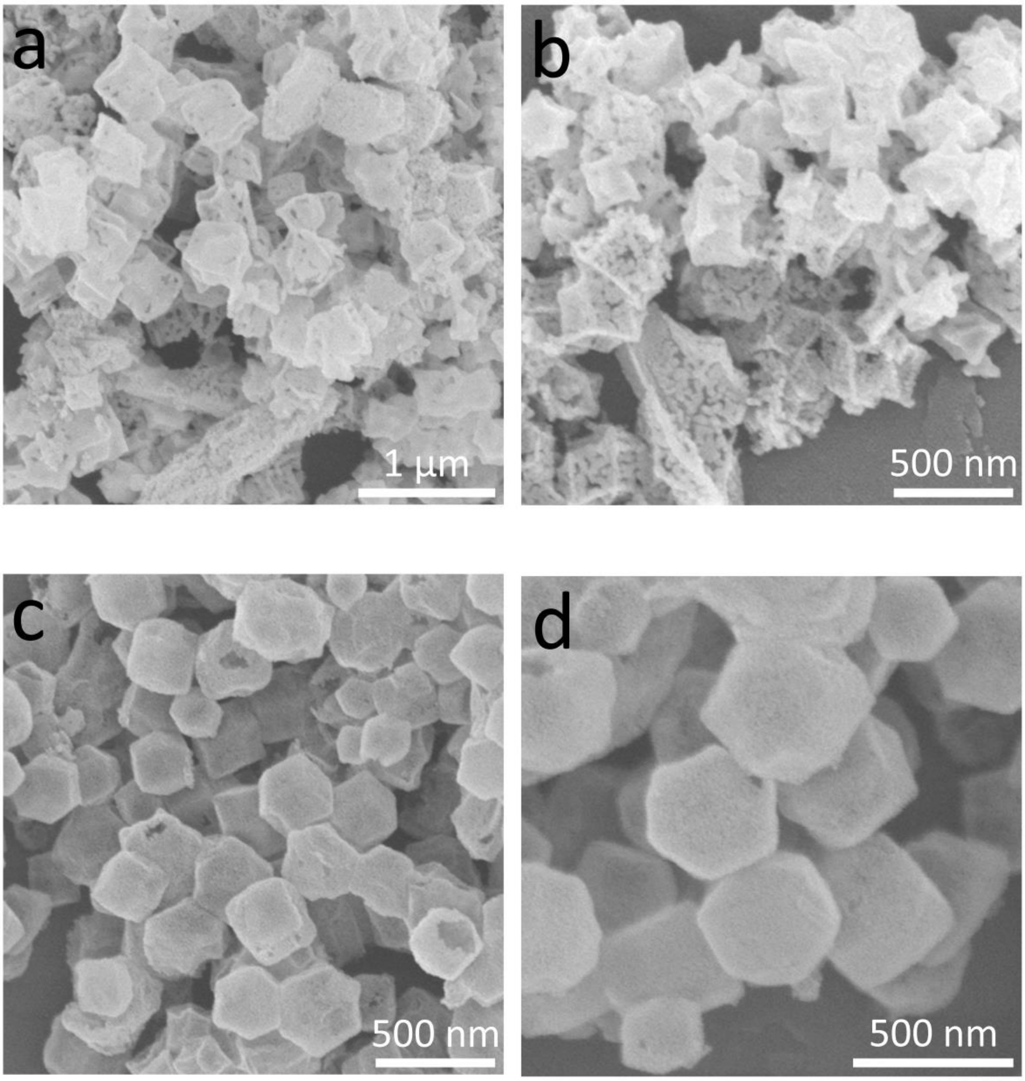

Figure S20. SEM images of (a, b) Co_3O_4 and (c, d) H-Co_3O_4.

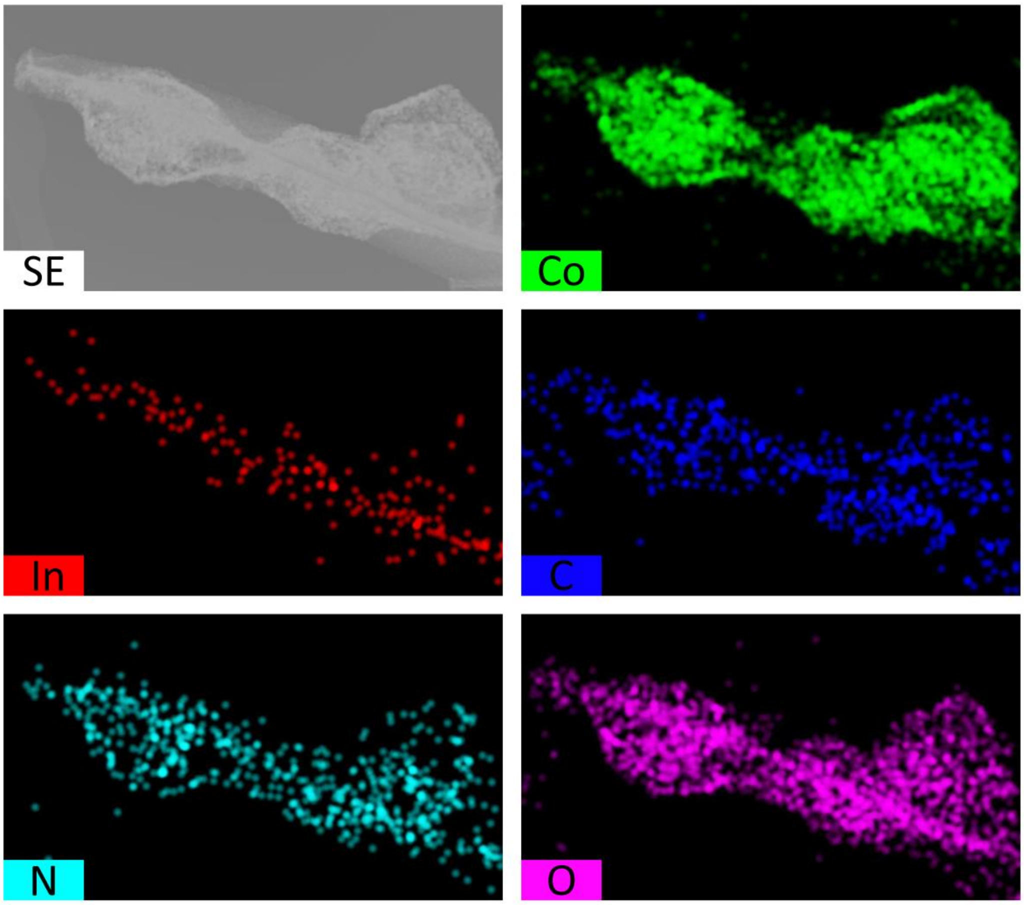

Figure S21. Elemental mappings of H-Co_3O_4/In_2O_3 with Co, In, C, N and O. Among them, the N element comes from the 2-methylimidazole ligand in ZIF-67 and the added TEA during the synthesis of InOF-1.

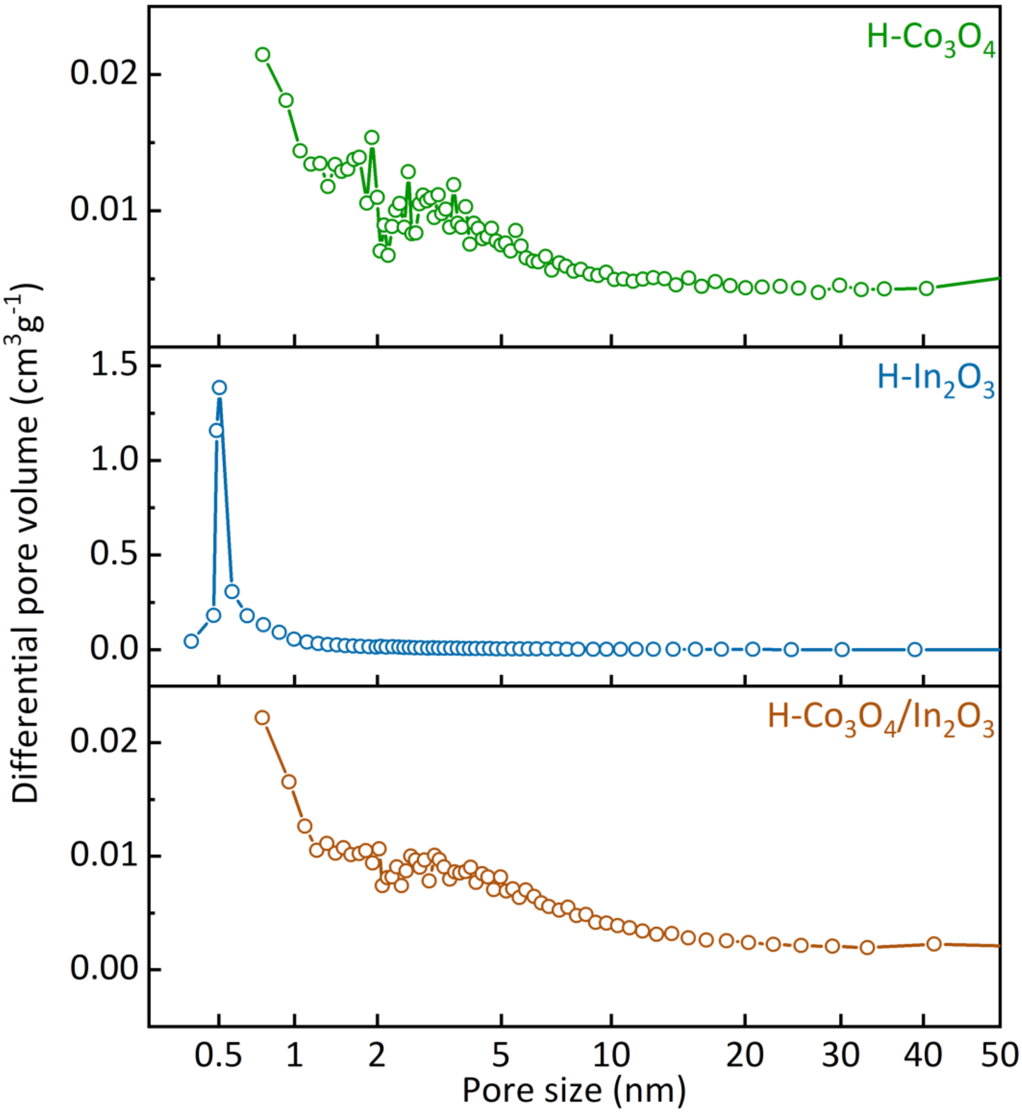

Figure S22. Pore size distribution curves of H-Co_3O_4, H-In_2O_3 and H-Co_3O_4/In_2O_3. H-Co_3O_4/In_2O_3 retains a similar pore size distribution to H-Co_3O_4, with a slightly increased pore volume due to the introduction of H-In_2O_3. All materials exhibit the coexistence of micropores and mesopores.

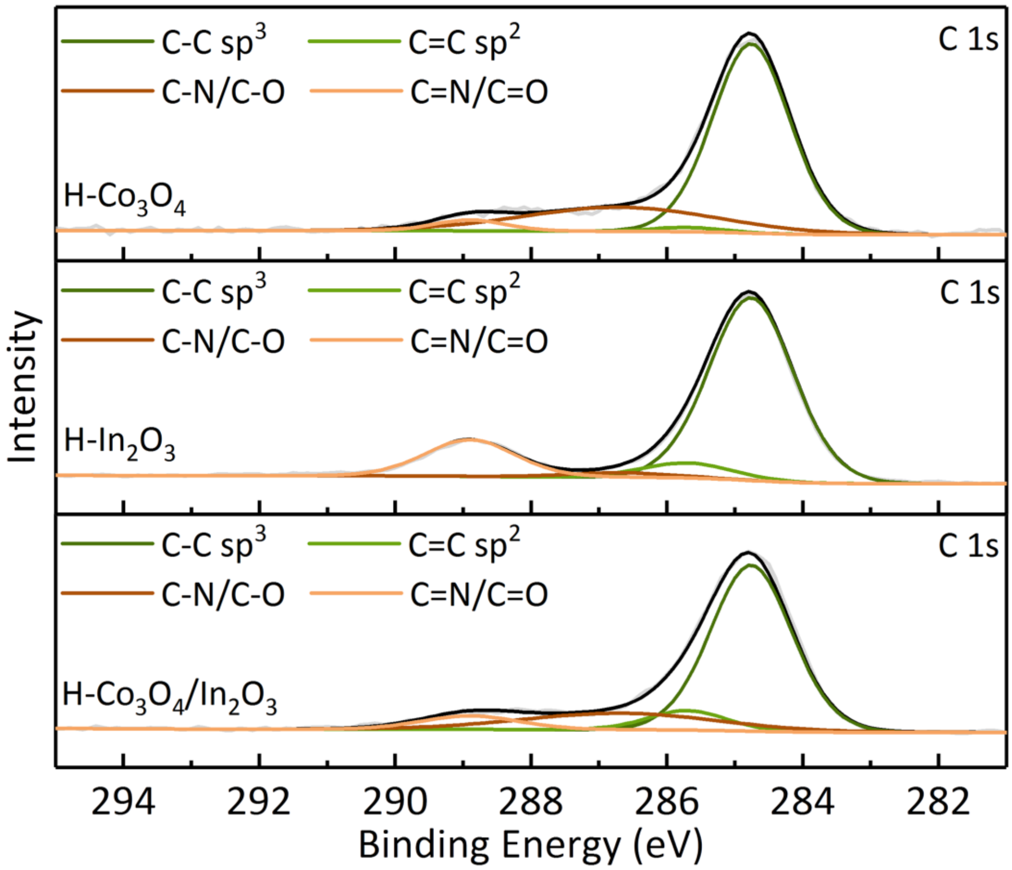

Figure S23. The deconvoluted spectra for C 1s of H-Co_3O_4, H-In_2O_3 and H-Co_3O_4/In_2O_3.

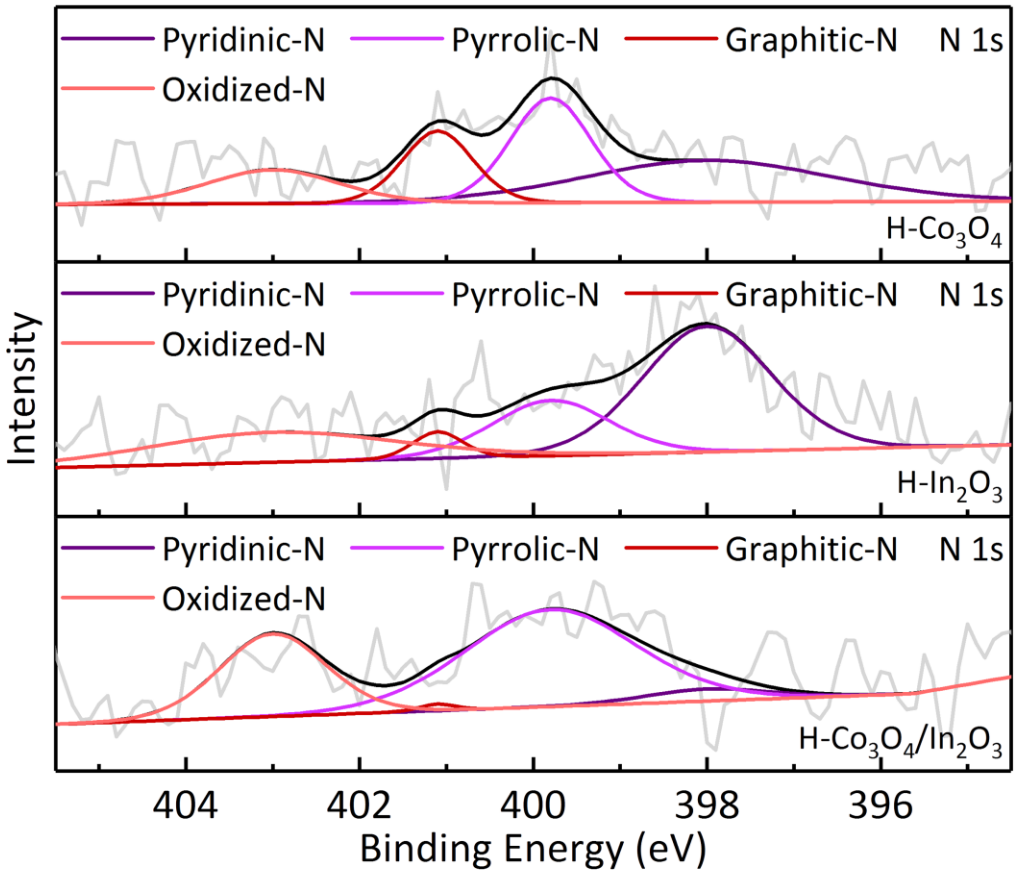

Figure S24. The deconvoluted spectra for N 1s of H-Co_3O_4, H-In_2O_3 and H-Co_3O_4/In_2O_3.

Table S1 Single Crystal X-Ray Data for Rod-like InOF-1[1].

Compound	**InOF-1**
CCDC	890421
Chemical formula	$In_2C_{16}H_{20}O_{16}$
Formula mass	696.41
Crystal system	Tetragonal
Space group	*I4₁22*
a (Å)	15.5665(6)
b (Å)	15.5665(6)
c (Å)	12.3201(1)
α	90°
β	90°
γ	90°
Unit cell volume (Å^3)	2985.36
Temperature (K)	293(2)
Z	8
F(000)	1328
No. of reflections measured	11697
R_{int}	0.024
Final R_1 values (I>2σ(I))	0.016
Final wR (F_2) values (I>2σ(I))	0.048

Table S2 Single Crystal X-Ray Data for ZIF-67[2].

Compound	**ZIF-67**
CCDC	671073
Chemical formula	$Co_3C_{24}H_{30}N_{12}O_2$
Formula mass	695.39
Crystal system	Cubic
Space group	*I-43m*
a (Å)	16.959(3)
b (Å)	16.959(3)
c (Å)	16.959(3)
α	90°
β	90°
γ	90°
Unit cell volume (Å^3)	4877.45(15)
Temperature (K)	153(2)
Z	4
F(000)	1420
No. of reflections measured	7062
R_{int}	0.0740
Final R_1 values ($I>2\sigma(I)$)	0.0724
Final wR (F_2) values ($I>2\sigma(I)$)	0.2138

Table S3. The Specific Surface Area and Pore Size Distribution Data of ZIF-67, InOF-1, ZIF-67-on-InOF-1 and Their Corresponding Etched Hollow Counterparts.

	Specific surface area (m^2 g^{-1})	Total pore volume (mL g^{-1})	Micro pore volume (mL g^{-1})	Average pore diameter (nm)
ZIF-67	1306.7497	0.7545	0.6763	2.3646
InOF-1	627.4320	0.3865	0.3174	2.4602
ZIF-67-on-InOF-1	932.4510	0.5774	0.4800	2.4958
H-ZIF-67	1292.5668	0.7527	0.6777	2.3309
H-InOF-1	733.9000	0.4364	0.3707	2.4069
H-ZIF-67-on-InOF-1	783.2374	0.4566	0.4159	2.3497

Table S4. The Specific Surface Area and Pore Size Distribution Data of Co_3O_4, In_2O_3, Co_3O_4/In_2O_3 and Their Corresponding Hollow Counterparts.

	Specific surface area (m^2 g^{-1})	Total pore volume (mL g^{-1})	Micro pore volume (mL g^{-1})	Average pore diameter (nm)
Co_3O_4	24.8368	0.2312	0.0069	35.2863
In_2O_3	302.9294	0.2018	0.1530	2.7214
Co_3O_4/In_2O_3	75.9613	0.3723	0.0260	21.0739
H-Co_3O_4	89.0778	0.6852	0.0333	33.8128
H-In_2O_3	474.3262	0.2910	0.2347	2.5232
H-Co_3O_4/In_2O_3	78.7612	0.3355	0.0315	19.7051

Table S5. The Deconvoluted O 1s, C 1s, N 1s, Co 2p, In 3d XPS Spectra of H-Co_3O_4, H-In_2O_3 and H-Co_3O_4/In_2O_3.

	M-O	C-O	C=O
H-Co_3O_4	529.9	531.3	532.5
H-In_2O_3	530.0	531.7	532.5
H-Co_3O_4@In_2O_3	530.0	531.4	532.5

	C-C sp^3	C=C sp^2	C-O/C-N	C=O/C=N
H-Co_3O_4	284.8	285.9	286.7	288.9
H-In_2O_3	284.7	285.7	286.7	288.9
H-Co_3O_4@In_2O_3	284.8	285.7	286.7	288.9

	Pyridinic-N	Pyrrolic-N	Graphitic-N	Oxidized-N
H-Co_3O_4	398.0	399.8	401.1	403.0
H-In_2O_3	398.0	399.8	401.1	402.9
H-Co_3O_4@In_2O_3	397.9	399.8	401.1	403.0

	Co^{3+} $2p_{3/2}$	Co^{2+} $2p_{3/2}$	Satellite-1	Co^{3+} $2p_{1/2}$	Co^{2+} $2p_{1/2}$	Satellite-2
H-Co_3O_4	779.6	781.0	790.0	794.7	796.4	804.6
H-Co_3O_4@In_2O_3	779.4	780.7	790.0	794.6	796.1	804.6

	In^{3+} $3d_{1/2}$	In^{3+} $3d_{3/2}$
H-In_2O_3	444.4	452.0
H-Co_3O_4@In_2O_3	444.6	452.2

Table S6. The Relevant Energy Data of H-Co_3O_4 and H-Co_3O_4/In_2O_3.

	E_{CB} (V vs. NHE)	E_{FB} (V vs. NHE)	E_g (eV)	E_{VB} (V vs. NHE)
H-Co_3O_4	1.17	0.97	1.77	-0.60
H-Co_3O_4/In_2O_3	1.13	0.93	1.86	-0.73

1. Jinjie Qian, Feilong Jiang, Daqiang Yuan, Mingyan Wu, Shuquan Zhang, Linjie Zhang, Maochun Hong, Highly selective carbon dioxide adsorption in a water-stable Indium-organic framework material. *Chem. Commun.*, **2012,** *48*, 9696-9698.

2. R. Banerjee, A. Phan, B. Wang, C. Knobler, H. Furukawa, M. O'Keeffe, O. M. Yaghi. High-Throughput Synthesis of Zeolitic Imidazolate Frameworks and Application to CO_2 Capture. *Science*, **2008**, 319, 939-943.
